# Mllt11 Regulates Migration and Neurite Outgrowth of Cortical Projection Neurons during Development

**DOI:** 10.1523/JNEUROSCI.0124-22.2022

**Published:** 2022-05-11

**Authors:** Danielle Stanton-Turcotte, Karolynn Hsu, Samantha A. Moore, Makiko Yamada, James P. Fawcett, Angelo Iulianella

**Affiliations:** ^1^Department of Medical Neuroscience, and Brain Repair Centre, Faculty of Medicine, Dalhousie University. Life Science Research Institute, Halifax, Nova Scotia B3H-4R2, Canada; ^2^Departments of Phamacology, Surgery, and Brain Repair Centre, Faculty of Medicine, Dalhousie University. Life Science Research Institute, Halifax, Nova Scotia B3H-4R2, Canada

**Keywords:** axonogenesis, callosal projection neurons, microtubules, Mllt11/Af1q, neuritogenesis, neurodevelopmental disorders

## Abstract

The formation of connections within the mammalian neocortex is highly regulated by both extracellular guidance mechanisms and intrinsic gene expression programs. There are two types of cortical projection neurons (CPNs): those that project locally and interhemispherically and those that project to subcerebral structures such as the thalamus, hindbrain, and spinal cord. The regulation of cortical projection morphologies is not yet fully understood at the molecular level. Here, we report a role for Mllt11 (Myeloid/lymphoid or mixed-lineage leukemia; translocated to chromosome 11/All1 Fused Gene From Chromosome 1q) in the migration and neurite outgrowth of callosal projection neurons during mouse brain formation. We show that *Mllt11* expression is exclusive to developing neurons and is enriched in the developing cortical plate (CP) during the formation of the superficial cortical layers. In cultured primary cortical neurons, Mllt11 is detected in varicosities and growth cones as well as the soma. Using conditional loss-of-function and gain-of-function analysis we show that Mllt11 is required for neuritogenesis and proper migration of upper layer CPNs. Loss of *Mllt11* in the superficial cortex of male and female neonates leads to a severe reduction in fibers crossing the corpus callosum (CC), a progressive loss in the maintenance of upper layer projection neuron gene expression, and reduced complexity of dendritic arborization. Proteomic analysis revealed that Mllt11 associates with stabilized microtubules, and *Mllt11* loss affected microtubule staining in callosal axons. Taken together, our findings support a role for Mllt11 in promoting the formation of mature upper-layer neuron morphologies and connectivity in the cerebral cortex.

**SIGNIFICANCE STATEMENT** The regulation of cortical projection neuron (CPN) morphologies is an area of active investigation since the time of Cajal. Yet the molecular mechanisms of how the complex dendritic and axonal morphologies of projection neurons are formed remains incompletely understood. Although conditional mutagenesis analysis in the mouse, coupled with overexpression assays in the developing fetal brain, we show that a novel protein called Mllt11 is sufficient and necessary to regulate the dendritic and axonal characteristics of callosal projection neurons in the developing mammalian neocortex. Furthermore, we show that Mllt11 interacts with microtubules, likely accounting for its role in neuritogenesis.

## Introduction

The mammalian neocortex is a complex structure, underlying the capacity for executive function, sensory processing, emotion, motor output, and cognition. Its laminated organization of excitatory pyramidal cortical projection neurons (CPNs) and inhibitory interneurons (INs) emerges from the coupling of neurogenesis with neuronal migration. Functional circuits emerge progressively during development as newborn neurons acquire molecular identities, dendritic morphologies, and projections characteristic of their laminar position. A fundamental question concerns how migration is controlled during sequential generation of more superficial cortical layers. While it is clear that the cytoskeleton plays a key role in neuronal translocation, its role in CPN subtype-specific neurite morphogenesis and the maintenance of laminar transcriptional programs is not well understood. The sorting of differentiating CPN subtypes into discrete layers occurs as cells migrate radially from progenitor domains populating the ventricular zone (VZ) and adjacent subventricular zone (SVZ) along the apicobasal axis to invade the cortical plate (CP). The earliest-born CPNs migrate along radial glial fibers projecting from the basal surface of VZ progenitors to the pia via somal translocation as forces exerted on the cytoskeleton by motor proteins push and pull the nucleus toward the pia until a neuron reaches its terminal location (Bellion et al., 2005; Tsai et al., 2007). As the somas of these cells approach the pia, the trailing apical pole becomes the nascent axon projecting to form white matter (WM) tracts (Nadarajah et al., 2001; Marín and Rubenstein, 2003; Ayala et al., 2007; Sakakibara et al., 2014). Consequently, the intermediate zone (IZ) and CP become densely populated, necessitating multiple modes of motility in later-born upper layer (UL). These events rely on coordinated cytoskeletal reorganization as cells transiently acquire multipolarity to weave through the dense IZ and then reacquire bipolarity on invasion of the CP (Sakakibara et al., 2014). The growth of axons during brain formation also involves dynamic cytoskeletal reorganization at the growth cone in response to external guidance cues or cell-cell contacts (Hirokawa and Takemura, 2004; Ayala et al., 2007).

The ability to couple developmental cues with construction or degradation of microtubule networks underlie the propensity of CPNs to form connections intracortically and extracortically. As such, mutations of cytoskeletal components and interactors have been linked to various neurodevelopmental disorders that affect cortical formation, stabilization, and projections at the level of mitosis, axon guidance, disturbance of fasciculation of axonal tracts, or impaired migration (Moon and Wynshaw-Boris, 2013; Moffat et al., 2015).

While axonogenic mechanisms are conserved across CPN subtypes, the targeting and wiring of each cortical layer is dependent on its unique molecular characteristics. Each cortical layer expresses a unique transcription factor program that provides the molecular instructions for synapse formation with appropriate target regions of the brain. Beginning with the earliest-born Cajal–Retzius (CR) cells of layer 1 (L1), which secrete Reelin to guide the radial migration of nascent neurons (Frotscher, 1998; Chai et al., 2009, 2016; Gil-Sanz et al., 2013), neurogenesis and lamination proceeds in an inside-out, cross-repressive manner. Transcription factors Tbr1 (Hevner et al., 2001; Thomson, 2010) and Bcl11b/Ctip2 (Arlotta et al., 2005) of deep layer (DL)6 and DL5, respectively, suppress UL-specific factors until DL formation is complete and subcerebral projections are initiated (Toma et al., 2014). UL neurogenesis follows with expression of Satb2 in thalamically-targeted L4 CPNs (Hisaoka et al., 2010; Leone et al., 2015; Lopez-Bendito and Molnar, 2003), and co-expression of Satb2 (Alcamo et al., 2008; Britanova et al., 2008; Leone et al., 2015), Cux1 (CDP), and Cux2 (Nieto et al., 2004; Zimmer et al., 2004) in UL2/3 corticocortical CPNs, 80% of which project across the corpus callosum (CC; Leone et al., 2015).

In an effort to identify regulators of neural migration and differentiation, we previously reported the expression of Mllt11/Af1q (Myeloid/lymphoid or mixed-lineage leukemia; translocated to chromosome 11/All1 Fused Gene From Chromosome 1q) in the developing CP (Yamada et al., 2014). Mllt11 is a vertebrate-specific, 90 amino acid protein first identified as a fused protein with Mll in infant acute myeloid leukemia (Tse et al., 1995). Here, we report that Mllt11 is a neural-specific tubulin-associating protein and its inactivation in the superficial layers of the mouse brain results in improper migration and formation of callosal projections. Loss of *Mllt11* led to neurite outgrowth defects coupled with loss of UL-specific transcriptional programs characteristic of neurodevelopmental disorders. In contrast, *Mllt11* overexpression promoted the invasion of the CP by differentiating neurons. We provide evidence that Mllt11 interacts with stabilized microtubules, consistent with *Mllt11* mutants phenocopying brain tubulinopathies (Bahi-Buisson et al., 2014). Altogether, these findings contribute significantly to our understanding of the genetic regulation of CPN development, dendritic complexity, and axonal connectivity.

## Materials and Methods

### Animals

All animal experiments were done according to approved protocols from the IACUC at Dalhousie University. Mice (*Mus musculus*) carrying a null mutation in the Mllt11 gene were generated using embryonic stem (ES) cell clones obtained from the mouse knock-out consortium project (UCDavis KOMP repository, *Mllt11tm1a(KOMP)Mbp*). The targeting construct is a “knock-out first, conditional second” approach, which inserts the gene encoding β*-galactosidase* (β*-gal*) into exon 2, the protein-coding sequence of the *Mllt11* gene. Two independently targeted clones were injected into blastocysts, and the resulting chimeras were mated to BL/6 females to achieve germ-line transmission. Offspring were genotyped by PCR using the following primers: wild-type forward (F) 5′-CGGTCCTGCCTTTGATTCTCAGC-3′ and reverse (R) 5′-GCCTACTGCACAAGGTTCTTCTTGG-3′ (expected product size: 379 bp), Mutant F 5′-GAGATGGCGCAACGCAATTAATG-3′ and R 5′-AAGCAGTATTTGCTTACTGGCCTGG-3′ (expected product size: 274 bp). Heterozygotes (*Mllt11tm1a(KOMP)Mbp/*+) were maintained on a C57BL/6 background and crossed with *FlpO*^+/−^ (*B6.Cg-Tg(Pgk1-flpo)10Sykr/J*, 011065, The Jackson Laboratory) mice and converted to a conditional allele via germ line Flp recombinase expression. Resulting *Mllt11^Flox/Flox^* offspring were crossed with *Ai9 Rosa26TdTomato* (B6.Cg-*Gt(ROSA)26Sor^tm9(CAG-tdTomato)Hze^*/J, 007909, The Jackson Laboratory) reporter line to generate *Mllt11^Flox/Flox^; Rosa26TdTomato*^+/−^ or *Mllt11^Flox/Flox^; Rosa26TdTomato*^+/+^ offspring.

*Cux2iresCre* mice used in this study to delete Mllt11 in developing UL neurons were previously described (Gil-Sanz et al., 2015; Yamada et al., 2015). *Cux2iresCre*^+/−^ mice (*B6(Cg)-Cux2*<*tm1.1(cre)Mull*>*/Mmmh*, Mutant Mouse Resource and Research Center) were crossed with *Ai9* (B6.Cg-*Gt(ROSA)26Sor^tm9(CAG-tdTomato)Hze^*/J, 007909, The Jackson Laboratory) mice. The resulting *Cux2iresCre*^+/−^*;Rosa26TdTomato*^+/−^ or *Cux2iresCre*^+/−^*;Rosa26TdTomato*^+/+^ offspring were crossed with the *Mllt11^Flox/Flox^; Rosa26TdTomato*^+/+^ line to conditionally knock out Mllt11. Offspring of these crosses were genotyped using the following primers: wild-type F 5′-CGGTCCTGCCTTTGATTCTCAGC-3′ and R 5′-GCCTACTGCACAAGGTTCTTCTTGG-3′ (expected product size: 379 bp), Post Flp/Cre F 5′-CGGTCCTGCCTTTGATTCTCAGC-3′ and R 5′AAGCAGTATTTGCTTACTGGCCTGG-3′, and Cre F 5′-GTTATAAGCAATCCCCAGAAATG-3′ and R 5′-GGCAGTAAAAACTATCCAGCAA-3′. We genotyped for the presence of TdTomato using primers F 5′-TACGGCATGGACGAGCTGTACAAGTAA-3′ and R 5′-CAGGCGAGCAGCCAAGGAAA-3′ (expected product size: 500 bp) and allelism was determined with primers F 5′-TCAATGGGCGGGGGTCGTT-3′, R 5′-TTCTGGGAGTTCTCTGCTGCC-3′, and R 5′-CGAGGCGGATCACAAGCAATA-3′ (expected product size: 250 pb for wild-type and 300 bp for mutant). Similar numbers of control and *Mllt11* cKO mutant male and female mouse embryos and neonates were used in the analysis. No sex differences in *Mllt11* cKO mutant phenotype were observed in pilot studies. Statistical analysis (see below) was conducted on combined numbers of male and female offspring.

### GST protein expression, mass spectrometry, and immunoprecipitation

#### GST-tagged protein expression

The entire *Mllt11* sequence was cloned into pGEX-4T-2 vector for GST pulldown assays. BL21 competent cells (Sigma) were transformed with pGEX-4T-2 (GST) and pGEX-4T-2-MLLT11 (GST-MLLT11). Colonies were picked and incubated overnight in 2-ml 2xYT media with ampicillin at 37°C with shaking. The following day, overnight culture was added to 150-ml 2xYT media with ampicillin and incubated at 37°C with shaking until OD_600_ reached 0.6. Protein expression was induced by adding IPTG (Invitrogen) to the culture to a final concentration of 0.1 mm and shaken at 28°C for ∼4 h. Cells were pelleted by centrifugation and resuspended in 8-ml NP-40 lysis buffer (50 mm Tris, pH 8.0, 150 mm NaCl, and 1% NP-40) with protease inhibitor cocktail (Sigma). Cells were nutated at 4°C for 10 min, sonicated on ice (30 s on/30 s off, for 3 min total, level 6 intensity), and again nutated at 4°C for 10 min. Lysed cells were pelleted and supernatant collected (protein lysate).

#### Immobilization of GST or GST-MLLT11 bait protein

GST or GST-MLLT11 proteins were immobilized to Glutathione Sepharose 4B beads (Pierce). GST or GST-MLLT11 protein lysates were added to equilibrated 50% bead slurry and nutated at 4°C for ∼3 h. Beads with immobilized protein were collected, washed, and resuspended in PBS to make a 50% slurry.

#### GST pull-downs and mass spectrometry

Whole embryonic mouse brains were harvested at embryonic day (E)15.5 and lysed in NP-40 lysis buffer with protease inhibitor cocktail (Sigma). Whole-brain lysates from one embryo were added to either 6-µg GST or 6-µg GST-MLLT11 bead slurries and nutated overnight at 4°C. Beads with bound lysate were pelleted, washed, resuspended in sample buffer. Samples were heated for 15 min at 37°C and run on an SDS-PAGE gel, then stained with Coomassie Blue (Pierce). Bands were cut from the SDS-PAGE gel and processed by Dalhousie's Biological Mass Spectrometry Core Facility.

#### Co-immunoprecipitation (co-IP) and Western blottings

HEK293 cells were transfected with myc or myc-MLLT11 vectors using Lipofectamine 2000 (Invitrogen). Twenty-four hours later, cells were lysed on ice with Tris-HCl lysis buffer containing 50 mm Tris-HCl, pH 7.4, 150 mm NaCl, 1 mm EDTA, 1% Triton X-100, and protease inhibitor cocktail (Sigma). Lysed cells were collected, centrifuged, and supernatant used for co-IP. Anti-c-Myc Agarose resin (Pierce) was used, as per manufacturer's protocol, to co-immunoprecipitate myc or myc-MLLT11 and their binding partners. Proteins were eluted from the resin with 50 mm NaOH, neutralized with 1 m Tris, pH 9.5, and added to nonreducing sample buffer for Western blot analysis. For whole-brain lysates, E18.5 cortical protein samples were separated on 8% SDS-PAGE gels for 1 h at 120 V and transferred overnight at 20 V on to PVDF membranes (Bio-Rad). Blots were probed with mouse anti-acetylated tubulin (1:20,000, Sigma), and rabbit anti-Mllt11 (1:2000, Abcam). Secondary antibodies were goat anti-rabbit HRP (1:5000, Invitrogen) and goat anti-mouse HRP (1:5000, Invitrogen). Blots were developed with Clarity Western ECL Substrate (Bio-Rad) and imaged on a ChemiDoc Touch Gel Imaging System (Bio-Rad). Band densitometry was done using Image Lab Software (Bio-Rad).

### qPCR

To confirm the loss of Mllt11 transcripts, E18.5 RNA was extracted from cortices of three genotypic conditional knock-outs (cKOs) and four controls using the RNeasy Micro kit (QIAGEN). RNA was reverse transcribed to cDNA using the SuperScript II Reverse Transcriptase kit (Invitrogen). qPCRs were conducted using the SensiFAST SYBR No-ROX kit (Bioline) with the following primers for Mllt11 and internal control GAPDH: Mllt11 F 5′-GAACTGGATCTGTCGGAGCT-3′ and R 5′-GCGCTCTCCAGAAGTTGAAG-3′, GAPDH F 5′-ACCACAGTCCATGCCATCAC-3′ and R 5′-TCCACCACCCTGTTGCTGTA-3′ (Weng et al., 2014). Reactions were performed in triplicates.

### Histology

Fetal brains were dissected out and fixed in 4% paraformaldehyde (PFA) for 4–8 h, depending on embryonic stage, before being equilibrated in sucrose, embedded in Optimum Cutting Temperature (OCT) compound (Tissue-Tek), and cryosectioned at 12 μm. Cortex morphology was assessed by DAPI staining. β-Gal staining was performed on cryosectioned slides using the β-Gal Tissue Stain kit (Millipore). Immunohistochemistry was conducted on E14.5–E18.5 brains as described previously (Iulianella et al., 2008).

Immunohistochemistry was conducted using the following antibodies: rabbit anti-CDP/Cux1 (1:100; Santa Cruz), mouse anti-Satb2 (1:250; Abcam), rat anti-Bcl11b/Ctip2 (1:500; Abcam), rabbit anti-Tbr1 (1:200; Abcam), rabbit anti-Cleaved Caspase 3 (CC3; 1:500; Cell Signaling Technology), rabbit anti-Pax6 (1:500; Abcam), goat anti-Sox2 (1:200; Santa Cruz), rat anti-Tbr2 (1:200; eBioscience), mouse anti-neurofilament 2H3 (1:200; DSHB, University of Iowa), rabbit anti-Tuj1 (Tubb3; 1:1000, Biolegend), and mouse anti-acetylated α-tubulin (1:1000, Sigma). Species-specific Alexa Fluor 488-, 568-, 594-, and/or 647-conjugated IgG (1:1500; Invitrogen) secondary antibodies were used to detect primary antibodies.

*In situ* hybridization was performed on 30-mm frozen sections obtained from E18.5, postnatal day (P)7, P14, P21, and P28 Control and cKO brains fixed overnight as previously described (Yamada et al., 2014) using an Mllt11 riboprobe.

For EdU birth dating studies, dams were injected intraperitoneally with 30 mg/kg body weight of EdU (Invitrogen) at E14.5, E16.5, and E18.5 and killed at E14.5 or E18.5. Sections were immunostained using the Click-It kit according to the manufacturer's protocol (Invitrogen).

For Golgi stains, brains were harvested from mice at P28 and subjected to the FD Rapid GolgiStain kit (FD Neurotechnologies) as per manufacturer instructions. Crude sections were cut and mounted on slides with Permount Mounting Medium (Fisher Scientific). Four brains were analyzed per genotype and 30 neurons per individual were analyzed.

#### Microscopy

Images were captured using a Zeiss AxioObserver fluorescence microscope equipped with an Apotome 2 structured illumination device, 10×, 20×, and a Hamamatsu Orca Flash v4.0 digital camera. β-Gal and *Mllt11 in situ* staining was captured using an upright Zeiss PrimoStar compound microscope with an ERc5s color camera. Images were processed using Zen software (Zeiss) and Photoshop CS6 (Adobe).

### Primary cortical cell culture and immunocytochemistry

Cortices were microdissected from E18.5 embryos, digested in trypsin (Pierce), manually triturated and plated on 35-mm well onto poly-D-lysine coated coverslips at a density of 150,000 cells for the neurite outgrowth assay or 500,000 for cellular localization experiments. Cells were plated in medium containing DMEM with 10% FBS and 1% penicillin/streptomycin and 4 h after plating, media was completely removed and replaced with Neurobasal media containing B-27+ (Gibco/Invitrogen), 1% penicillin/streptomycin, and L-glutamine. Cells were cultured for 24 h or one week in a 37°C incubator containing 5% CO_2_, then fixed for 10 min in 4% paraformaldehyde. Immunocytochemistry was conducted using the following antibodies: mouse anti-Tau (1:200; Abcam), rabbit anti-MAP2 (1:1000; Abcam), rabbit anti-Mllt11 (1:300, Abcam), rabbit anti-Tuj1 (Tubb3; 1:1000, Biolegend), and mouse anti-acetylated α-tubulin (1:1000, Sigma).

### *In utero* electroporation and cDNA constructs

*In utero* electroporation was conducted using standard methodology under sterile surgical conditions (Saito, 2006). Endotoxin-free DNA was prepared according to the instructions of the manufacturer (QIAGEN) and injected at 1.5 µg/µl into the telencephalic vesicles of embryos in time-staged pregnant females anesthetized under inhalable isoflurane (5 l/min). A small incision was made on the ventral midline of anesthetized pregnant FVB females under a sterile field treatment. Single uteri containing the E13.5 fetuses were extruded and electric current was delivered across the fetal brains as five pulses for 50 ms at 900-ms intervals using tweezer-style electrodes linked to the pulse generator CUY21 Vivo SQ (Sonidel). The embryos were returned in the body cavity, the peritoneum was sutured and the skin was stapled. Experimental plasmids used were *Mllt11-ires-eGFP*, and control plasmids included *pIRES2-EGFP* (Clonetech) or pCIG (Addgene). To ensure comparable development staging, for each dam one uterus was electroporated with the experimental construct and the other with the control vector. Fetuses were allowed to survive for 2 d until E15.5 after which they were processed for cryosectioning to evaluate GFP expression and cortical layer development by immunostaining. *N* = 3 Mllt11-eGFP and *N* = 4 eGFP control fetal brains were analyzed.

### DiI tracing of callosal projections

Brains of E18.5 embryos were removed and embedded in 7% low gelling temperature agarose in DMEM medium with 1% penicillin/streptomycin. Embedded brains were crudely sectioned at a thickness of ∼1–2 mm. Approximately 0.5 µl of DiI (Invitrogen) was injected into the cortical WM tracts at rostral and caudal axial levels and incubated for 8 h in Tyrode's solution. Tissues were then fixed overnight at 4°C in 4% paraformaldehyde before incubating for two weeks at room temperature in PBS with 1% penicillin/streptomycin. Images were captured using a Zeiss V16 Axiozoom fluorescent stereomicroscope equipped with an Axiocam 506 mono digital camera (Zeiss).

### Image sampling, quantification, and analysis

For analysis of immunostaining markers and EdU, counting frames (100 × 100 µm) were placed in a vertical strip along the somatosensory (S1) cortex with the first counting frame along the edge of the ventricle. At least three histologic sections within the somatosensory cortex from three to eight different animals were analyzed for each immunostain, EdU or electroporated vector. Cells that were positively labeled for both DAPI and the marker were counted within each frame using ImageJ (FIJI; Schindelin et al., 2012). For analysis of primary cortical cell culture and Golgi stain, neurites were traced and measured and Sholl analyses were conducted using the “simple neurite tracer” plugin in FIJI. For analysis of radial glial parameters, E14.5, E16.5, and E18.5 cortical slices were immunostained with goat anti-Nestin antibodies (1:250, Santa Cruz). Control and *Mllt11* cKO mutant images were captured with identical acquisition parameters and radial glial morphology was assessed using the “directionality” plugin in FIJI to calculate the percentage of fibers in the image aligned in the same direction. Automatic thresholds were then applied and the area covered by Nestin staining was measured and expressed as a percentage of the total image area (Extended Data Fig. 3-1).

To ensure consistency among samples, cell counts were restricted to the presumptive S1 (somatosensory cortex) of the embryonic brain. In all experiments, three to six Mllt11 KO and control embryos were used for quantification analysis using the unbiased and systematic sampling method we described previously (Yamada et al., 2015). Counts for Ctip2, Tbr1, Cux1, Tbr2, Sox2, Pax6, CC3, and Satb2 are represented as line graphs, quantifying the proportion of DAPI stained cells expressing those markers. For EdU, TdTomato, and DAPI distribution analyses, proportion of total stained cells expressing markers within each cortical bin was quantified to ensure counting methods were consistent with previous studies (Chen et al., 2008; Lawrenson et al., 2017; Reyes et al., 2019). Image montages were assembled using Photoshop CS6.

### Statistical analysis

Statistical differences were determined with Student's *t* tests (two-tailed) with Welch's correction. Sholl analyses were performed with paired *t* tests (two-tailed) to compare branch points within comparable radii. Bar charts, line graphs, and statistical testing were conducted using GraphPad Prism V5.0d software, with results shown as mean ± SD. In all quantification studies, significance level was set at *p* ≤ 0.05 (*p ≤ 0.05, **p ≤ 0.01, ***p ≤ 0.001, ****p ≤ 0.0001).

## Results

### cKO of *Mllt11* from UL2/3 CPNs

**Figure 1.**
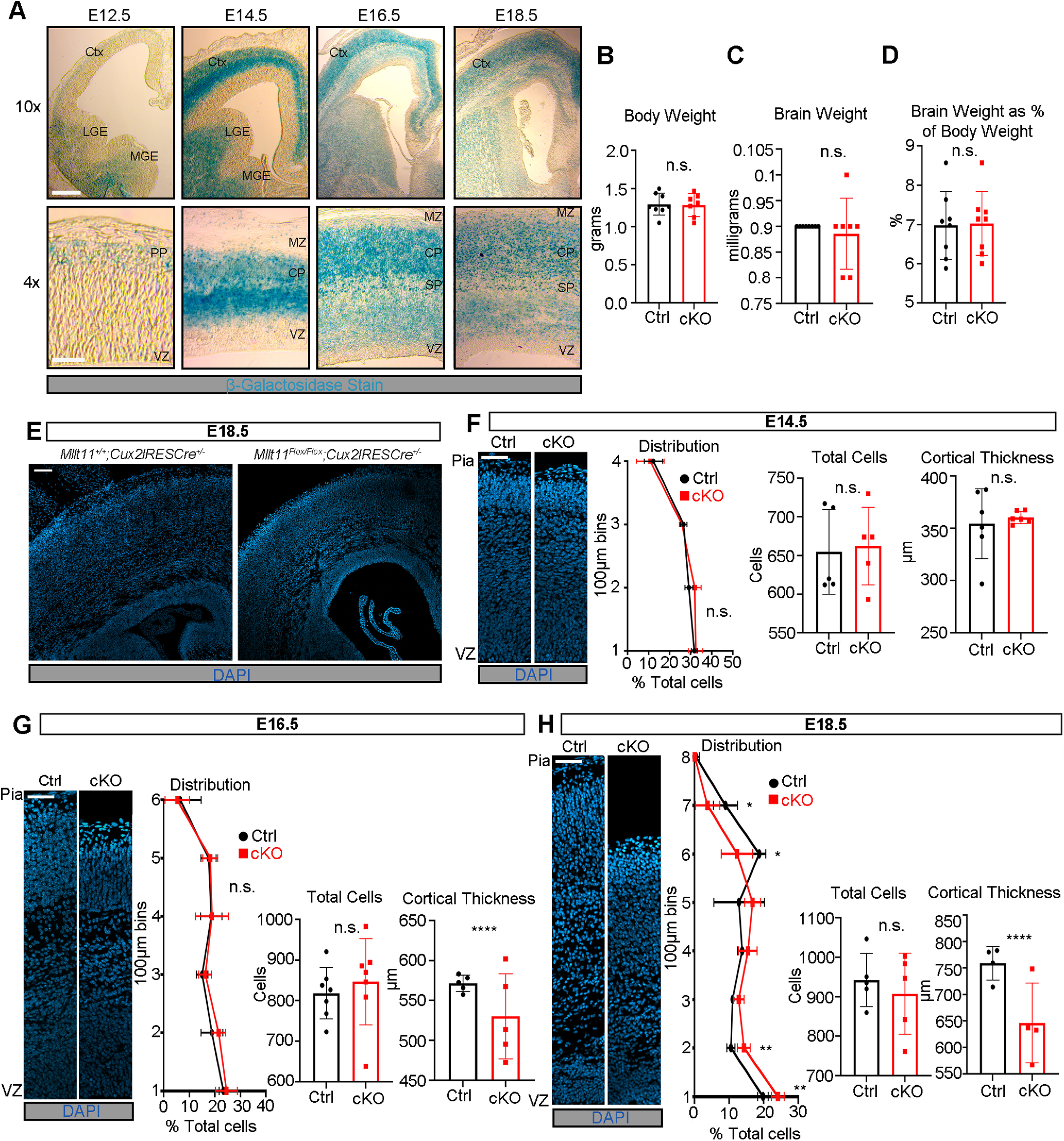
*Mllt11* is expressed in the developing CP and *Mllt11* loss affects the laminar distribution of cells. ***A***, Coronal sections of the targeted *Mllt11* locus (which inserted a *lacZ* cDNA) showing *Mllt11* expression across four time points during cortical neurogenesis through β-Gal staining. At E14.5, β-Gal expression was most intense in the CP, corresponding to UL neurogenesis. By E16.5, β-Gal staining intensity shifted to the superficial cortex where UL CPNs were accumulating. ***B–D***, Body weight (***B***), brain weight (***C***), and brain weight as a percentage of body weight (***D***) showed no difference between control (cntl) and cKOs at E18.5. ***E***, cKO cortices were thinner than controls at E18.5. ***F–H***, Thinning of the cKO cortex was progressive, with thicknesses being comparable to controls at E14.5 (***F***), but *Mllt11* mutants exhibited reduced thickness and reduced distribution of cells in the superficial cortex at E16.5 (***G***), which increased in severity by E18.5 (***H***). Total cell counts and cortical thickness measurements (µm) are shown as bar graphs. Cell count line charts represent percentage of positive cells normalized to DAPI+ nuclei per 100 × 100 μm bin. Student's *t* test with Welch's correction, (***B–D***) *N* = 8, (***F***) *N* = 5, (***G***) *N* = 7 for total cells, *N* = 5 for cortical thickness, (***H***) *N* = 5 for total cells, *N* = 4 for cortical thickness. Data presented as mean ± SD n.s., not significant; *p ≤ 0.05, **p ≤ 0.01, ***p ≤ 0.001, ****p ≤ 0.0001. Scale bars: 100 μm (***A***), 100 μm (***E***), 50 μm (***F–H***). Ctx, cortex; CP, cortical plate; IZ, intermediate zone; LGE, lateral ganglionic eminence; MGE, medial ganglionic eminence; MZ, marginal zone; PP, preplate; SP, subplate; VZ, ventricular zone. See Extended Data Figures 1-1, 1-2, 1-3, 1-4, 1-5.

Mllt11 is expressed in developing neurons of the central nervous system, including the neocortex, but its role in cortical neurogenesis is unknown (Yamada et al., 2014). To investigate the role of Mllt11 in neurogenesis, we generated mice carrying a null mutation in the *Mllt11* gene using ES cell clones obtained from the mouse knock-out consortium project (UCDavis KOMP repository, *Mllt11^tm1a(KOMP)Mbp^*). We used the targeted (unflipped) allele to visualize *Mllt11* by β-galactosidase (β-gal) staining (Fig. 1*A*) because of the *LacZ* gene inserted into the *Mllt11* locus (Extended Data Fig. 1-1*A*). Initial *Mllt11* locus activity begins in the pallial MZ at E12.5, showing stronger expression in the IZ of the fetal cortex at E14.5 coinciding with the birth of basal progenitors fated to become UL2/3 CPNs, intensifying in the superficial cortical layers by E16.5–E18.5 (Fig. 1*A*). The localization of β-Gal staining reflected the activity of the *Mllt11* locus and confirmed our previous report of *Mllt11* mRNA and protein localization in differentiating neurons of the neocortex (Yamada et al., 2014). A detailed examination of *Mllt11* transcript expression by *in situ* hybridization revealed that cortical expression was maintained until P21, when it began to taper to levels indistinguishable from background at P28 (Extended Data Fig. 1-1*B*,*C*). In the hippocampus, *Mllt11* transcript levels decreased progressively from P7 to P21 and remained low at P28 (Extended Data Fig. 1-1*C*).

10.1523/JNEUROSCI.0124-22.2022.f1-1 Extended Data Figure 1-1 *Mllt11* targeting strategy and ontogenic expression profile. ***A***, Graphic representation of the targeting construct inserted into the *Mllt11* locus before and after flp and cre recombinase activity. The entire protein-coding region of *Mllt11* is encoded by exon 2 (Mllt11 ex2), which is flanked by loxP sites. *Mllt11* expression can be evaluated by β-Gal staining because of the insertion of *lacZ* cDNA in the targeted allele. A cKO allele can be garneted by the removal of the *lacZ* and selection cassette by germline Flp recombination. ***B***, A reference coronal section of a control brain with a boxed region to indicate the area sampled in panel ***C***. ***C***, *Mllt11* expression in the cortex (top panel) and hippocampus (bottom panel) from P7 to P28. RNA levels declined in the cortex were indistinguishable from background at P28. Scale bar: 100 μm. Download Figure 1-1, TIF file.

**Figure 2.**
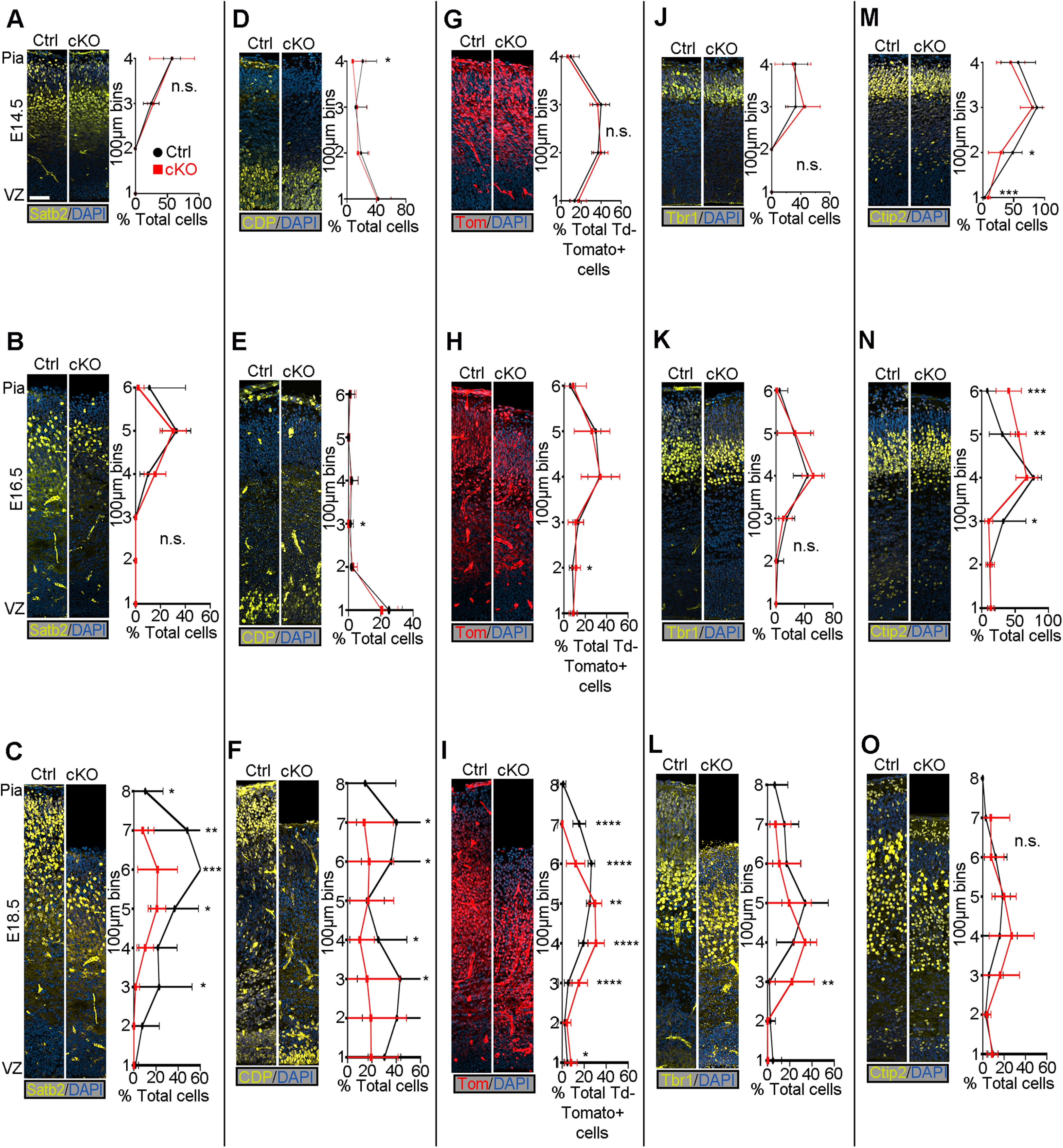
*Mllt11* loss progressively perturbed the formation of UL CPNs. ***A–O***, Coronal cortical slices stained for cortical layer markers. ***A***, ***B***, Coronal cortical slices stained for Satb2 expression were similar between *Mllt11* cKOs and controls at E14.5 (***A***) but displayed decreased numbers of Satb2+ cells in upper bins, and an apical shift at E16.5 (***B***). ***C***, *Mllt11* cKO cortices displayed decreased Satb2+ cell numbers and apical shift at E18.5 compared with controls. ***D***, ***E***, CDP/Cux1+ cell numbers were largely normal at E14.5 (***D***), but began to decrease at E16.5 (***E***). ***F***, CDP/Cux1 levels were severely decreased in *Mllt11* cKOs compared with controls at E18.5. ***G–I,*** Distribution of *TdTomato* expression in the cortex was comparable between controls and cKOs at E14.5 (***G***), E16.5 (***H***), and E18.5 (***I***). ***J–L***, Tbr1 levels and localization were comparable between control and cKO at E14.5 (***J***) and E16.5 (***K***), but its expression domain exhibited an apical shift at E18.5 (***L***). ***M–O***, Ctip2+ cell numbers and localization were largely unaltered in cKO at E14.5 (***M***) and E16.5 (***N***) but exhibited an apical shift in expression domain at E18.5 (***O***). Line charts represent percentage of positive cells normalized to DAPI+ nuclei per 100 × 100 μm bin (***A–F***, ***J–O***) and percentage of total TdTomato+ cells per 100 × 100 μm bin (***G–I***). Student's *t* test with Welch's correction, (***A***, ***E***, ***J***, ***L–O***) *N* = 4, (***F–I***) *N* = 5, (***G***) *N* = 5 controls, 6 mutants, (***K***) *N* = 4 controls, 5 mutants. Data presented as mean ± SD n.s., not significant; *p ≤ 0.05, **p ≤ 0.01, ***p ≤ 0.001, ****p ≤ 0.0001. Scale bar: 50 μm (***A–O***). VZ, ventricular zone. UL CPNs, upper layer cortical projection neurons. See Extended Data Figure 2-1.

10.1523/JNEUROSCI.0124-22.2022.f1-2 Extended Data Figure 1-2 *Mllt11* cKO validation by qPCR and ISH. ***A***, Quantitation of qPCR fluorescence levels of control and cKO cortices normalized to internal control GAPDH. ***B***, Fold change of *Mllt11* cDNA transcript levels was significantly decreased in cKO relative to control brains. ***C***, ***D***, Images of ISH of *Mllt11* riboprobe on P7 control and cKO cortices (***C***) and hippocampi (***D***) showed decreased labeling in the superficial cortex, corresponding to the Cux2-expressing region. Student's *t* test with Welch's correction, (***A***, ***B***) *N* = 4 controls, 3 cKOs, (***C***, ***D***) *N* = 3. Data presented as mean ± SD; **p ≤ 0.01. Scale bar: 100 μm. CP, cortical plate; RFU, relative fluorescence units. Download Figure 1-2, TIF file.

**Figure 3.**
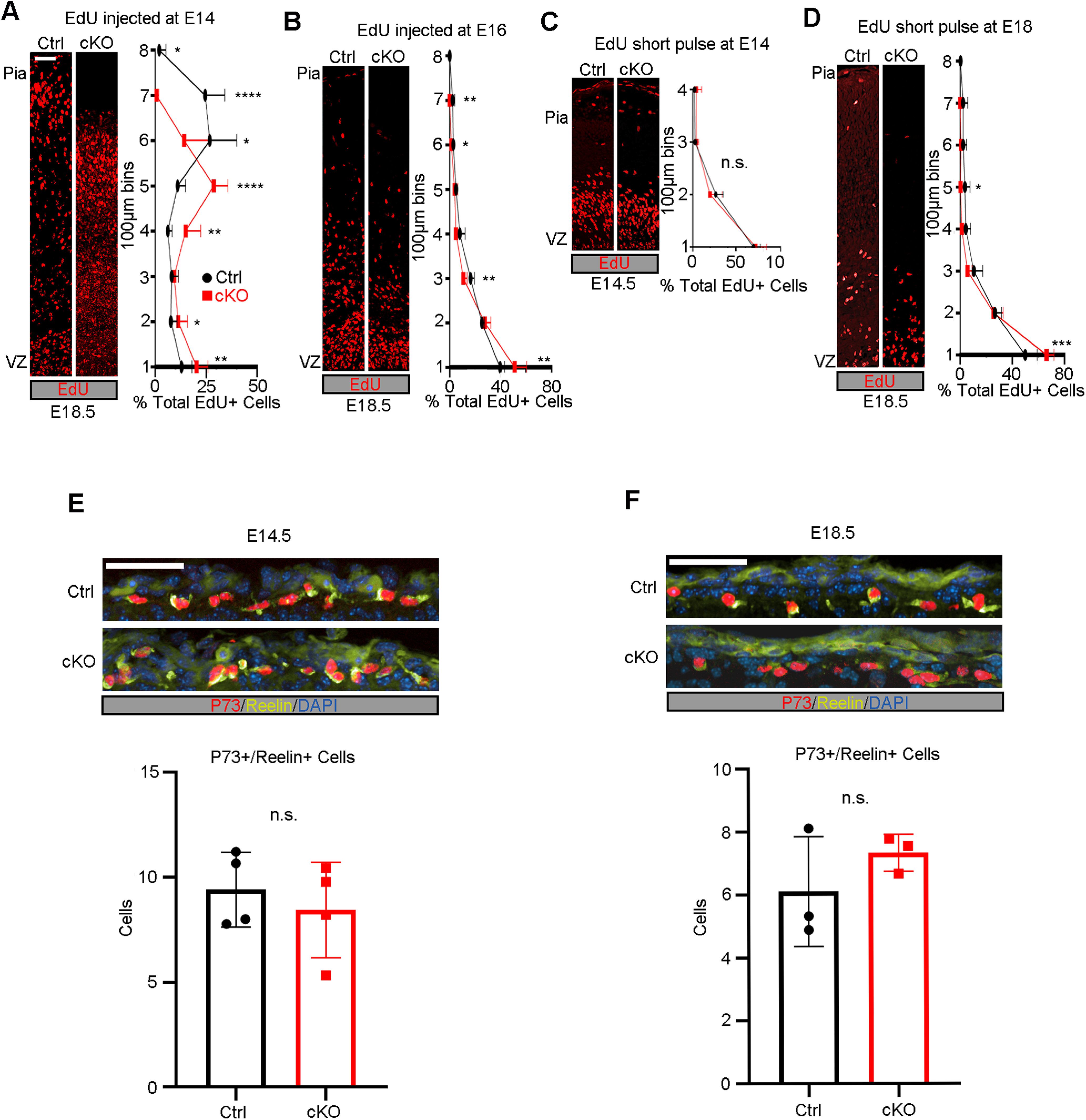
EdU birthdating demonstrated a CPN migratory defect in *Mllt11* cKO mutants. ***A***, ***B***, EdU birthdating of UL CPNs by injection at E14 showed altered distribution in *Mllt11* cKOs at E18.5, reflecting an apical shift in expression correlating with the decreased cortical thickness. ***B***, Apical shift in migrating CPNs in cKOs relative to controls following an EdU pulse at E16 (***B***). ***C***, Proliferating cells labeled with a short pulse of EdU at E14 showed no significant differences in numbers or distribution between controls and cKOs. ***D***, No significant difference in numbers of nuclei labeled by a short pulse of EdU at E18 populating most bins in cKOs versus controls, but a greater proportion of cells were retained in the cKO VZ region. ***E***, ***F***, Pial sections at E14.5 (***E***) and E18.5 (***F***) showed comparable levels of expression of CR markers P73 and Reelin in L1 of cKOs versus controls. Line charts represent percentage of positive cells per 100 × 100 μm bin as a proportion of total EdU+ cells. Total co-labeled cells are shown as bar graphs. Student's *t* test with Welch's correction, (***A***, ***C***, ***E***) *N* = 4, (***B***, ***D***, ***F***) *N* = 3. Data presented as mean ± SD n.s., not significant; *p ≤ 0.05, **p ≤ 0.01, ***p ≤ 0.001, ****p ≤ 0.0001. Scale bar: 50 μm (***A–F***). VZ, ventricular zone. CPN, cortical projection neuron. See Extended Data Figure 3-1.

10.1523/JNEUROSCI.0124-22.2022.f1-3 Extended Data Figure 1-3 Loss of *Mllt11* had no impact on programmed cell death. ***A***, ***B***, Cortical slices at E14.5 (***A***) and E18.5 (***B***) showed no differences in the levels of CC3 in cKOs relative to controls. ***C***, CC3 staining in the retrosplenial area, which normally has enhanced apoptosis, was included as an antibody control for CC3 staining. White arrowheads indicate positive labeling. *N* = 3 controls, 5 cKOs. Scale bar: 50 μm. VZ, ventricular zone. Download Figure 1-3, TIF file.

**Figure 4.**
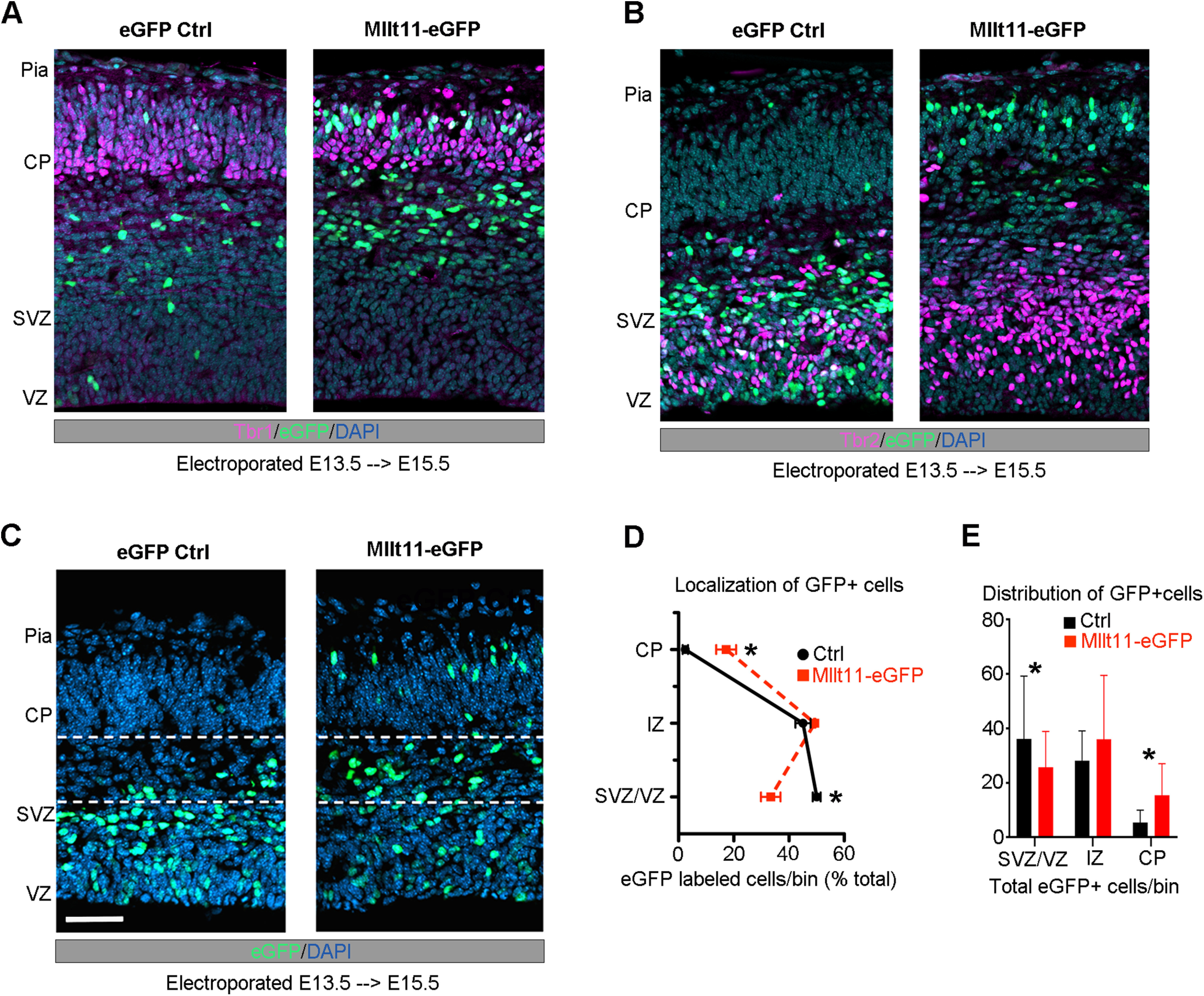
*Mllt11* overexpression promoted migration into the CP. ***A***, ***B***, E15.5 coronal cortical sections after electroporation at E13.5 wither either a control eGFP (left) or *Mllt11-ires-eGFP* (Mllt11-eGFP) bicistronic plasmid (right). ***B***, *Mllt11-eGFP* electroporation promoted migration into the CP, identified by Tbr1 staining. ***C***, Control GFP (eGFP-control) electroporated cells remained mostly within the SVZ/VZ, identified by Tbr2 staining. ***D***, ***E***, Localization of eGFP-control and Mllt11-eGFP+ cells quantified as a percentage of total eGFP+ cells (***D***), and as the distribution of total eGFP+ cells per fetal cortical layer bin (***E***). Student's *t* test with Welch's correction; *N* = 3 Mllt11-eGFP, *N* = 4 eGFP controls. Data presented as mean ± SD; *p ≤ 0.05. Scale bar: 25 μm (***A***). CP, cortical plate; IZ intermediate zone; SVZ, subventricular zone; VZ, ventricular zone.

10.1523/JNEUROSCI.0124-22.2022.f1-4 Extended Data Figure 1-4 Neural progenitor populations were largely unaffected in *Mllt11* cKO mutants. ***A–C***, Coronal cortical slices stained for Pax6 with IHC was unaltered in cKO cortices relative to controls at E14.5 (***A***), E16.5 (***B***), and E18.5 (***C***). ***D–F***, Sox2 levels were largely similar between cKO and controls at E14.5 (***D***), E16.5 (***E***), and E18.5 (***F***). ***G–I***, Tbr2 expression was also largely unaltered in cKOs at E14.5 (***G***) and E16.5 (***H***), but showed a significant trend toward decreased levels normalized to DAPI+ nuclei immediately above the Tbr2+ progenitor domain at E18.5 (***I***). Line charts represent percentage of positive cells normalized to DAPI+ nuclei per 100 × 100 μm bin. Student's *t* test with Welch's correction, (***A–C***, ***F–I***) *N* = 4, (***D***, ***E***) *N* = 3. Data presented as mean ± SD n.s., not significant; *p ≤ 0.05, **p ≤ 0.01, ***p ≤ 0.001. Scale bar: 50 μm (***A–I***). VZ, ventricular zone. Download Figure 1-4, TIF file.

**Figure 5.**
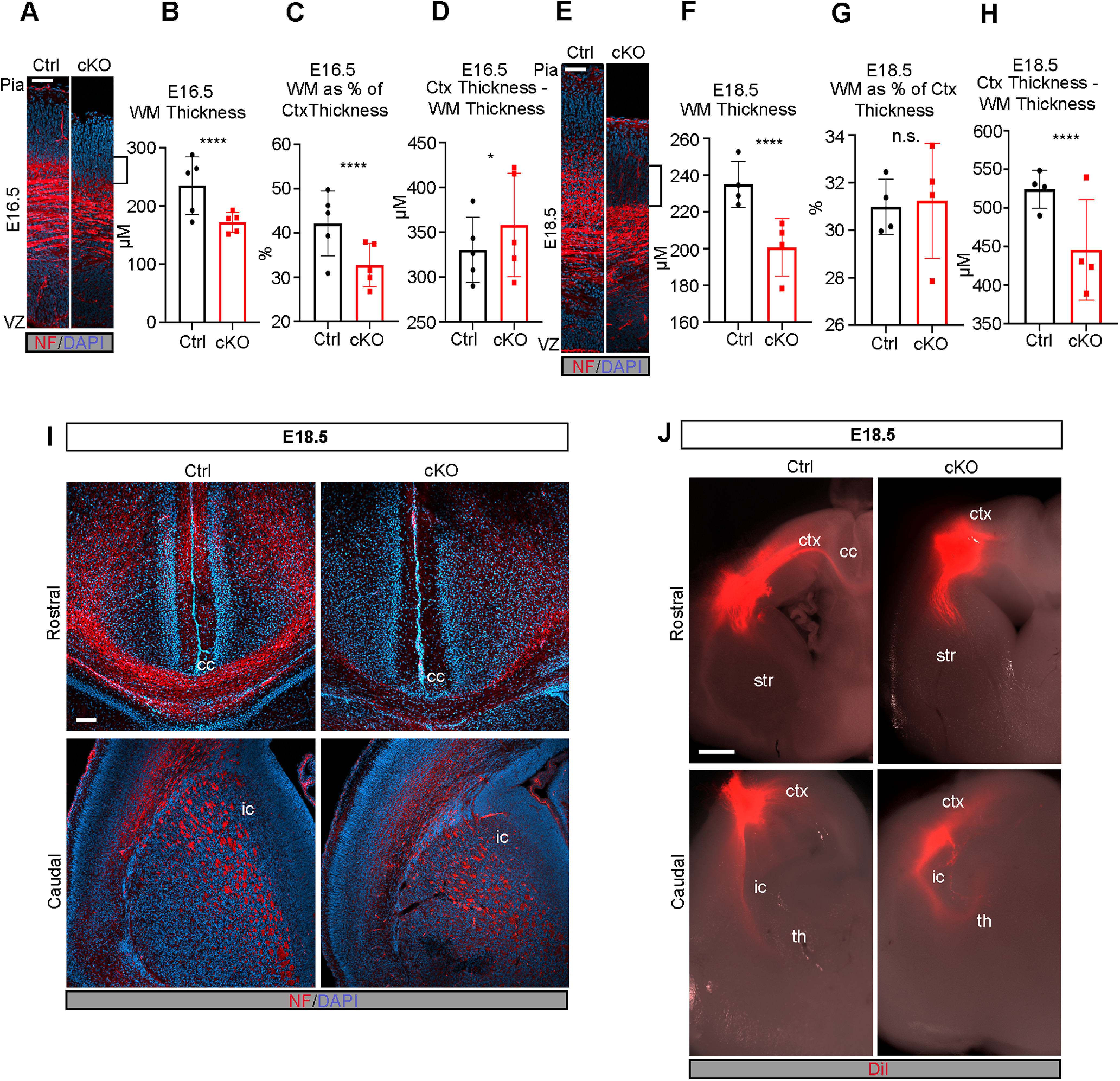
Formation of WM tracts and callosal projections is impaired in the *Mllt11* cKO cortex. ***A***, Image of cortical WM tracts labeled with neurofilament at E16.5. Brackets indicate decreased WM. ***B***, ***C***, Quantification of cortical WM thickness (***B***) and WM thickness as a proportion of total cortical thickness (***C***) showed significant decrease in WM in cKOs relative to controls at E16.5. ***D***, Quantification of cortical thickness subtracted from WM tract thickness showed a slight increase in *Mllt11* cKO mutants compared with controls at E16.5. ***E***, Image of cortical WM tracts labeled with neurofilament at E18.5. Brackets show decreased WM staining area. ***F–H***, Quantification of cortical WM thickness (***F***), WM thickness as a proportion of total cortical thickness (***G***), and cortical thickness subtracted from WM tract thickness (***H***) all showed significant decreases in *Mllt11* mutants compared with controls at E18.5. ***I***, ***J***, Coronal sections of E18.5 cortices at rostral (upper panels) and caudal (lower panels) axial levels labeled with neurofilament (***I***) or traced with DiI (***J***). ***I***, Neurofilament labeling of the corpus callosum was significantly decreased in cKO compared with controls but labeling of the internal capsule was unaffected. ***J***, DiI labeling was absent in the corpus callosum of cKO slices while control cortices displayed crossing fibers labeled by DiI. Rostrally, the internal capsule was traced comparably in control and cKO cortices. Student's *t* test with Welch's correction, (***A–D***) *N* = 5, (***E–H***) *N* = 4, (***J***) *N* = 3. Data presented as mean ± SD; ****p ≤ 0.0001. Scale bar: 200 μm (***A***) and 50 μm (***B***). ctx, cortex; cc, corpus callosum; str, striatum; th, thalamus; ic, internal capsule; VZ, ventricular zone; WM, white matter.

10.1523/JNEUROSCI.0124-22.2022.f1-5 Extended Data Figure 1-5 Total cell numbers of neuronal progenitors in control and *Mllt11* cKO cortices. ***A–C***, Distribution of total Pax6+ cells in control (black lines) and *Mllt11* cKO (red lines) at E14.5 (***A***), E16.5 (***B***), and E18.5 (***C***) showing comparable levels at all stages. ***D–F***, Distribution of total Sox2+ cells at E14.5 (***D***), E16.5 (***E***), and E18.5 (***F***) showing comparable levels at all stages. ***G–I***, Distribution of total Tbr2+ cells at E14.5 (***G***), E16.5 (***H***), and E18.5 (***I***) showing comparable levels at E14.5 (***G***) and E16.5 (***H***), and slightly decreased levels immediately above the Tbr2+ progenitor domain in cKOs at E18.5 (***I***). Line charts represent total cells positive for each marker per 100 × 100 μm bin. Student's *t* test with Welch's correction, (***A–C***, ***F–I***) *N* = 4, (***D***, ***E***) *N* = 3. Data presented as mean ± SD n.s., not significant; *p ≤ 0.05, **p ≤ 0.01, ***p ≤ 0.001. Download Figure 1-5, TIF file.

Given the temporal and spatial overlap between *Mllt11* expression and neurogenic regions of the cerebrum, we set out to investigate the role of Mllt11 in the development cerebral cortex, and specifically in the formation of the UL2/3 callosally projecting CPNs that bridge communication interhemispherically (Fame et al., 2011) where *Mllt11* expression appeared highest during corticogenesis (Fig. 1*A*). We used a targeted *Mllt11* null allele to generate cKO *Mllt11* mice, in which the entire protein coding sequence in Exon 2 is flanked by loxP sites (Extended Data Fig. 1-1*A*). To avoid nonspecific effects of the selection cassette, mice encoding germline expressing flp recombinase were crossed to mice harboring the *Mllt11* targeted allele, leaving only loxP sites used to excise the entire protein-coding exon (exon 2) to create a conditional allele. Subsequently, to inactivate the *Mllt11* in UL2/3 of the cerebral cortex, we crossed the *Mllt11^Flox/Flox^* mice to the *Cux2iresCre* strain along with the *Ai9 TdTomato* reporter mice to visualize recombined neurons, creating *Mllt11* cKO mutant (cKO) fetuses and neonates. Cux2 is highly expressed in developing UL2/3 projections neurons, and mosaically expressed in the SVZ in the fetal pallial cortex (Nieto et al., 2004; Zimmer et al., 2004; Cubelos et al., 2008a,b). We confirmed *Mllt11* loss in UL CPNs by qPCR of E18.5 cortices (Extended Data Fig. 1-2*A*,*B*) as well as by ISH at P7 (Extended Data Fig. 1-2*C*,*D*) from *Cux2iresCre*-driven *Mllt11* cKO brains.

### Loss of *Mllt11* leads to a progressive decrease in cortical thickness

Brain and body weight did not differ significantly between *Mllt11* cKO mutants and controls at E18.5 (Fig. 1*B–D*). Mutants displayed a thinning of the cortex visible in the CP and WM beneath it (Figs. 1*E*, 5*A*,*E*). To explore the origin of this cortical thinning, we examined the morphology of the cortex beginning at E14.5 (Fig. 1*F*) when UL CPNs are born (Zimmer et al., 2004; Cubelos et al., 2008b). A thinning of the mutant cortex was observed beginning at E16.5 (Fig. 1*G*), increasing in severity by E18.5 (Fig. 1*H*). This was reflected by significant changes in the distribution of DAPI+ nuclei in deeper bins of the *Mllt11* cKOs, with a larger proportion of DAPI+ nuclei localized apically (toward the VZ) in E18.5 mutants relative to controls (Fig. 1*H*). We also evaluated whether programmed cell death could account for the cortical thinning, but did not find any cells positive for the apoptotic marker CC3 in the *Mllt11* cKO mutant neonatal cortex (Extended Data Fig. 1-3*A*,*B*), while retrosplenial regions, which typically show enhanced apoptosis, showed similar CC3+ staining in both controls and mutants (Extended Data Fig. 1-3*C*).

*Mllt11* expression was restricted to the developing CP and not in the apical progenitor regions (VZ) of the cortex (Fig. 1*A*). To rule out potential cell nonautonomous contributions from neural progenitors to the *Mllt11* mutant cortical phenotype we quantified cells positive for progenitor markers from E14.5 to E18.5, corresponding to the period of UL neurogenesis. Pax6, a radial glial cell (RGC) marker (Götz et al., 1998), was unaltered on *Mllt11* loss at all observed time points (Extended Data Figs. 1-4*A–C*, 1-5*A–C*). Sox2, a less restrictive marker for neural progenitors and quiescent RGCs (D'Amour and Gage, 2003; Ellis et al., 2004), also exhibited no significant differences in expression in the *Mllt11* cKO mutants compared with controls (Extended Data Figs. 1-4*D–F*, 1-5*D–F*). Tbr2+ intermediate (basal) progenitors, which give rise to UL 2/3 CPNs, did not differ markedly between control and *Mllt11* cKO groups, but displayed fewer cells within deep (nonprogenitor) regions of the mutant cortex at E18.5, suggesting alterations in the formation and/or migration of nascent neurons from the SVZ (Extended Data Figs. 1-4*G–I*, 1-5*G–I*).

### *Mllt11* is required for the maintenance of UL CPN molecular identity

The transcriptional de-repression loop that specifies cell types in discrete cortical laminae coincides with the birth and migration of projection neurons (Kumamoto et al., 2013; Toma et al., 2014). Thus, a loss of *Mllt11* in UL progenitors can potentially affect neuronal birth, migration, and/or specification. We therefore investigated whether *Mllt11* loss had any role in regulating the molecular identity of UL CPNs. Expression of Satb2 in CPNs of L2-4 exhibited a decrease at E18.5 (Fig. 2*C*; Extended Data Fig. 2-1*C*) as did CDP/Cux1, another marker of UL CPNs (Fig. 2*F*; Extended Data Fig. 2-1*F*). Investigation at earlier developmental time points revealed that this phenotype was progressive, beginning at E16.5 and increasing in severity by E18.5 (Fig. 2*A*,*B*,*D*,*E*; Extended Data Fig. 2-1*A*,*B*,*D*,*E*). While the extent of CDP/Cux1 and Satb2 staining displayed variability across individual mutants, there was a consistent decrease in expression levels an apical shift in expression domain in all *Mllt11* cKOs (Fig. 2*C*,*F*; Extended Data Fig. 2-1*C*,*F*). TdTomato labeling was used as a short-term lineage tracer of Cux2+ intermediate progenitors that give rise to UL2/3 CPNs, and while TdTomato levels were largely unaltered at all time points (Fig. 2*G–I*), there was a consistent apical shift in expression domain at E18.5 (Fig. 2*I*). Collectively, these findings suggested that *Mllt11* loss in nascent UL neurons did not affect their neurogenesis, or the activation of UL gene expression programs, but may have affected their migration to contribute to L2/3 formation.

10.1523/JNEUROSCI.0124-22.2022.f2-1 Extended Data Figure 2-1 Total cell numbers of cortical layer markers in control and *Mllt11* cKO cortices. ***A–C***, Distribution of total Satb2+ cells in control (black lines) and *Mllt11* cKO (red lines), with comparable levels at E14.5 (***A***) and E16.5 (***B***), but a significant decrease in the mutant cortex at E18.5 (***C***). ***D–F***, Distribution of total CDP+ cells with comparable levels at E14.5 (***A***) and E16.5 (***B***) and a decrease in mutants compared at E18.5 (***F***). ***G–I***, Distribution of total Tbr1+ cells in the cortex showing comparable levels at E14.5 (***G***) and E16.5 (***H***), with mutants exhibiting an apical shift in expression domain at E18.5 (***I***). ***J–L***, Distribution of total Ctip2+ cells in the cortex showing comparable levels at E14.5 (***J***), and an apical shift in expression domain beginning at E16.5 (***K***) and persisting at E18.5 (***L***) in *Mllt11* cKO mutants compared with controls. Line charts represent total cells positive for each marker per 100 × 100 μm bin. Student's *t* test with Welch's correction, (***A***, ***E***, ***G***, ***I–L***) *N* = 4, (***F***) *N* = 5, (***H***) *N* = 4 controls, 5 mutants. Data presented as mean ± SD n.s., not significant; *p ≤ 0.05, **p ≤ 0.01, ***p ≤ 0.001. Download Figure 2-1, TIF file.

The cortical de-repression loop functions on a cellular level by alternately repressing DL and UL fate (Toma et al., 2014) so we wanted to determine whether the loss of UL CPN fate was coupled with a complementary upregulation of DL CPN fates in these cells. We examined the localization of DL6-specific and DL5-specific markers Tbr1 and Ctip2, respectively, in *Mllt11* cKOs and controls at E14.5–E18.5 (Fig. 2*J–O*; Extended Data Fig. 2-1*G–L*). *Mllt11* cKO mutants displayed an apical shift in the expression domains of both Tbr1 and Ctip2 from E16.5 to E18.5, consistent with decreased cortical thickness and impaired UL formation (Fig. 2*K–O*; Extended Data Fig. 2-1*H–L*).

### *Mllt11* is required for the migration of UL CPNs

Given the thinning of the cortex and shift of cortical layer markers throughout development, we hypothesized that Mllt11 may regulate CPN migration into the CP. To explore the role of *Mllt11* in CPN migration, we pulsed pregnant dams with EdU to label UL CPNs at E14 or E16 and observed the localization of EdU at E18.5. CPNs labeled with EdU at E14 exhibited an apical shift of roughly 100 µm in the *Mllt11* cKO mutants relative to controls (Fig. 3*A*), consistent with a decrease in cortical thickness in the cKOs (Figs. 1, 2). A greater proportion of E14-born CPNs remained restricted to the deeper bins of *Mllt11* cKO cortices relative to controls (Fig. 3*A*). As CPNs labeled with EdU at E16 were still in transit by E18, with only a few control CPNs reaching the outermost layers of the cortex, the discrepancy in UL cell migration was less pronounced in cKOs (Fig. 3*B*). To examine whether *Mllt11* loss affected the neurogenesis of UL neurons, we pulsed dams with EdU for 2 h at E14, which efficiently labeled nuclei within the basal progenitor region (Fig. 3*C*). We observed no difference in the numbers or distribution of EdU+ nuclei in *Mllt11* mutants versus control cortices after a short pulse at E14 (Fig. 3*C*). To determine whether *Mllt11* loss caused a delay in the timing of UL neurogenesis, we repeated this short pulse at E18 when UL neurogenesis is nearly complete and noted no differences in EdU+ cells in the superficial cortex of *Mllt11* mutant versus control brains (Fig. 3*D*). We did observe a slight increase of EdU+ cells in the deepest 100-µm bin of the *Mllt11* mutant cortex relative to controls, possibly reflecting aberrant migration of later born neurons (Fig. 3*D*). Overall, the EdU birth dating analysis suggested that Mllt11 primarily regulates the migration, but not neurogenesis, of UL CPNs into the CP.

10.1523/JNEUROSCI.0124-22.2022.f3-1 Extended Data Figure 3-1 Cortical radial glia were unaltered in *Mllt11* cKOs. ***A***, Coronal cortical slices at E14.5 showing Nestin immunostainiong in control versus *Mllt11* cKO mutants. ***B–D***, Representative angle (***B***), area (***C***), and fiber dispersion (***D***) showed no significant differences between controls and cKOs. ***D***, Nestin expression in the cortex at E16.5. ***E–G***, Representative angle (***E***), area (***F***), and dispersion (***G***) showed no significant differences between control and cKO cortices at E16.5. ***I***, Nestin staining at E18.5. ***J–L***, Representative angle (***J***), area (***K***), and fiber dispersion (***L***) show no significant differences between controls and cKOs at E18.5. Student's *t* test with Welch's correction, (***A–D***) *N* = 4, (***E–H***) *N* = 5, (***I–L***) *N* = 3. Data presented as mean ± SD n.s., not significant. Scale bar: 50 μm (***A***, ***E***, ***I***). VZ, ventricular zone. Download Figure 3-1, TIF file.

Guidance of migratory CPNs is dependent on the earliest born CR cells of L1, which secrete Reelin to attract and halt neuronal migration at the pial interface (Tueting et al., 1999; Hirota and Nakajima, 2017). However, there were no differences in the expression of CR markers p73 or Reelin at E14.5 (Fig. 3*E*) or E18.5 (Fig. 3*F*) following *Mllt11* loss in UL progenitors and nascent neurons, suggesting that the apparent migration defect was not because of abrogated Reelin signaling. Additionally, *Mllt11* loss did not result in the perturbation of the radial glial scaffold on which migrating nascent neurons rely to reach their respective laminae, as evidenced by the comparable distribution of Nestin+ radial glia fibers in the *Mllt11* cKO mutant and control fetal cortices (Extended Data Fig. 3-1*A*,*E*,*I*). We quantified the radial glial angle (Extended Data Fig. 3-1*B*,*F*,*J*), average area covered (Extended Data Fig. 3-1*C*,*G*,*K*), and dispersion of fibers (Extended Data Fig. 3-1*D*,*H*,*L*) at E14.5, E16.5, and E18.5, respectively, and found no significant differences between controls and cKOs. We conclude that the migratory defects in *Mllt11* mutant cortices were not likely caused by alterations in the radial glial scaffold.

Given the migration deficit in *Mllt11*mutants, we examined whether its overexpression in fetal brains is sufficient to promote migration to the superficial regions of the developing cortex. We used *in utero* electroporation to express a bicistronic vector containing *Mllt11* and *GFP* cDNAs, or control *GFP* vector alone, in wild-type mouse embryonic forebrains. We performed electroporations in anesthetized pregnant dams at E13.5, by injecting a DNA solution containing expression vectors unilaterally into forebrain ventricles, and analyzed the resulting fetal brains 2 d after at E15.5, when neurons are migrating into the CP. At E15.5, neurons from fetal brains overexpressing *Mllt11-GFP* localized to the CP, demarcated with Tbr1 staining (Fig. 4*A*,*D*,*E*), while neurons from fetal brains electroporated with GFP-expressing control plasmids had yet to migrate out of the SVZ and IZ, identified by Tbr2 staining (Fig. 4*B–E*). This experiment demonstrated that *Mllt11* overexpression is sufficient to promote migration into the developing CP. Taken together, our loss and gain-of-function analyses strongly support a cell autonomous role for Mllt11 in promoting nascent UL neuronal migration in the developing CP.

### *Mllt11* loss leads to reduced callosal crossing fibers and a thinning of WM tracts

The migratory defects alone may be insufficient to explain the decreased cortical thickness of *Mllt11* cKO brains at E18.5 (Fig. 1*E*,*H*). Given that reduction in UL CPNs (Fig. 2*A*,*B*) would likely affect interhemispheric projections (Aboitiz and Montiel, 2003; Alcamo et al., 2008; Fame et al., 2011), we examined whether *Mllt11* loss impacted formation of projection fibers contributing to the cortical WM tracts by staining for NF in coronal brain slices. We observed notable decreases in the thickness of NF+ fibers in the region of the fetal cortex containing WM tracts at E16.5 (Fig. 5*A–D*) and E18.5 (Fig. 5*E–H*) following *Mllt11* loss. Importantly, while NF+ fiber staining in the cc was greatly reduced in *Mllt11* cKOs at E18.5, NF+ fibers in the internal capsule, reflecting corticothalamic projections, remained unchanged in the mutant brains (Fig. 5*I*). Taken together, our findings revealed a critical role for Mllt11 in UL CPN neurite morphology and axonogenesis of callosal fibers connecting the developing cerebral hemispheres.

The reduction of NF+ fibers crossing the cc in mutant brains suggested that *Mllt11* loss impacted the ability of UL2/3 CPNs to extend fibers across the telencephalic midline. To address the potential inability of *Mllt11* cKOs to extend axonal projections, we injected the lipophilic fluorescent dye DiI into the WM tracts of cortical slices at E18.5 and allowed it to diffuse through the tissue for two weeks before visualization. DiI labeling of projections was not detectable in the cc of *Mllt11* cKOs, while controls exhibited clearly labeled crossing callosal fibers (Fig. 5*J*). In contrast, the corticothalamic projections of the internal capsule were intact and traced by the DiI in both controls and cKOs, demonstrating that corticothalamic projections from DL6 were unaffected by the *Cux2iresCre* driven *Mllt11* cKO strategy (Fig. 5*J*). Altogether, our findings demonstrated that *Mllt11* loss in UL neurons specifically impacted the development of callosal projections.

### Mllt11 interacts with cytoskeletal proteins

**Figure 6.**
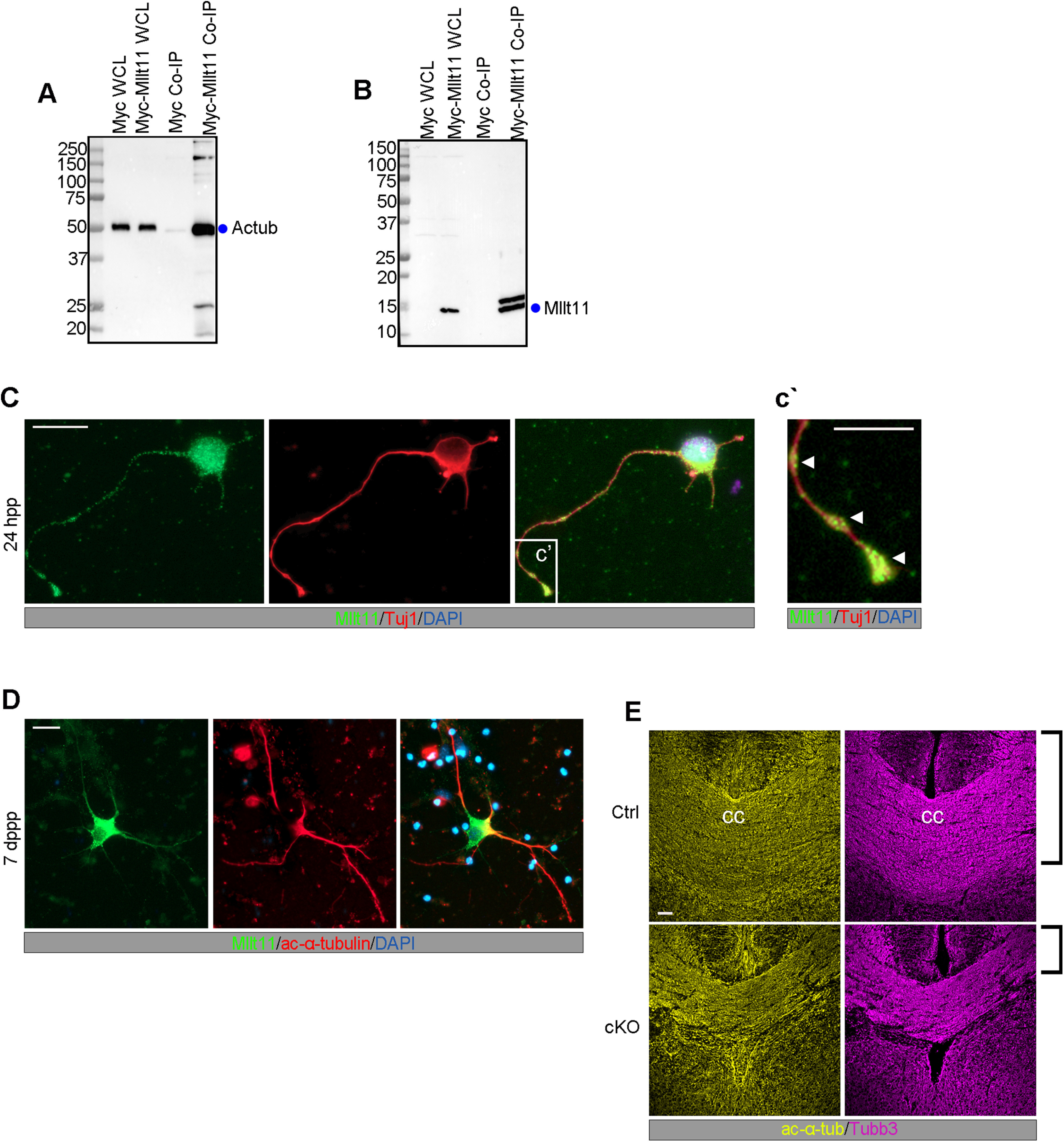
Mllt11 associated and colocalized with acetylated α-tubulin in growing neurites. ***A***, Co-IP of acetylated α-tubulin (Actub) with Myc-tagged Mllt11 in whole cell lysates of HEK293 cells compared with a myc programmed control. ***B***, Co-IP probed with Mllt11 antibody showing IP signal restricted to lanes containing Mllt11. ***C***, ***D***, Primary cortical neurons cultured for 24 h (***C***) or 7 d (***D***) postplating. ***C***, After 24 h, Mllt11 colocalizes with Tubb3 in the growth cone and at swellings along the distal axon as indicated by arrowheads (***c'***), and in the soma (***C***). After 7 d, Mllt11 was primarily found along proximal portions of the axon where it colocalizes with acetylated α-tubulin (***D***). ***E***, Coronal sections of the corpus callosum (cc) at E18.5 labeled with acetylated α-tubulin and Tubb3. Brackets show decreased thickness in cKO compared with control. Student's *t* test with Welch's correction, (***A***, ***B***) *N* = 4 (controls and cKOs). Data presented as mean ± SD n.s., not significant (i.e., *p* > 0.05). Scale bar: 20 μm (***C***, ***D***) and 50 μm (***E***).

Our birthdating analysis suggested that the migratory defects observed in *Mllt11* cKOs are likely independent of the maintenance of UL transcriptional programs. To identify potential pathways through which Mllt11 might be exerting its effects on CPN migration, we performed a GST pulldown assay to identify interacting proteins using lysate from E18.5 brains. Potential Mllt11-interacting proteins are listed in Table 1 and include multiple α and β tubulin isoforms (>70% coverage) as well several atypical myosins, such as Myosin 5a (36%), Myosin 9 (24%), and Myosin 10 (8%). A potential association between Mllt11 and tubulin is consistent with a recent whole cell proteomic study, which identified Mllt11 as a likely interactor of both α-tubulins and β-tubulins (Go et al., 2021). Given that acetylation of α-tubulin is associated with stabilized microtubules and is crucial for outgrowth of stable neurites, we decided to focus on validating interactions between Mllt11and acetylated α-tubulin by immunoprecitiation analysis. We overexpressed a myc-tagged Mllt11 in Hek293 cells and pulled down acetylated-α-tubulin (Fig. 6*A*). Probing the blots with anti-Mllt11 antibodies confirmed this interaction was dependent on heterologous Mllt11 expression in the cells (Fig. 6*B*), validating an association between Mllt11 and stabilized tubulin isoforms.

**Table 1.** Potential Mllt11-interaction targets from GST pull-downs in E18.5 whole-brain lysates

Band	Accession	Description	Sum PEP score	Coverage (%)	# Peptides	# PSMs	# AAs	MW (kDa)	Reference
7	D3Z4J3	Unconventional myosin-Va (Myo5a)	201.776	36	53	240	1855	215.4	
7	Q8VDD5	Myosin-9 (Myh9)	181.542	24	36	142	1960	226.2	
6	Q3UH59	Myosin-10 (Myh10)	31.272	8	10	22	2013	233.3	
5	P63017	Heat shock cognate 71-kDa protein OS = *Mus musculus* OX = 10090 GN = Hspa8 PE = 1 SV = 1	199.247	59	32	303	646	70.8	Li et al. (2014)
5	P38647	Stress-70 protein, mitochondrial (Hspa9)	20.395	12	6	12	679	73.4	
5	P20029	Endoplasmic reticulum chaperone BiP (Hspa5)	23.74	10	5	50	655	72.4	
5	Q3THK7	GMP synthase [glutamine-hydrolyzing] (Gmps)	8.67	5	3	6	693	76.7	
5	O08553	Dihydropyrimidinase-related protein 2 OS = *Mus musculus* OX = 10090 GN = Dpysl2 PE = 1 SV = 2	12.297	15	4	7	572	62.2	
4	Q9CWF2	Tubulin β-2B chain (Tubb2b)	381.396	74	29	1338	445	49.9	
4	Q7TMM9	Tubulin β-2A chain (Tubb2a)	380.755	74	29	1340	445	49.9	
4	P99024	Tubulin β-5 chain (Tubb5)	369.155	74	29	1232	444	49.6	
4	P68372	Tubulin β-4B chain (Tubb4b)	365.578	74	29	1153	445	49.8	
4	Q9ERD7	Tubulin β-3 chain (Tubb3)	347.246	72	28	970	450	50.4	
4	Q9D6F9	Tubulin β-4A chain (Tubb4a)	324.979	73	27	993	444	49.6	Go et al. (2021)
4	P68369	Tubulin α-1A chain (Tuba1a)	255.603	66	27	557	451	50.1	Go et al. (2021)
4	P05213	Tubulin α-1B chain (Tuba1b)	241.479	66	26	519	451	50.1	
4	Q922F4	Tubulin β-6 chain (Tubb6)	176.166	40	17	444	447	50.1	
4	P10126	Elongation factor 1-α1 (Eef1a1)	74.451	37	10	62	462	50.1	
4	A2AQ07	Tubulin β-1 chain (Tubb1)	54.274	13	7	202	451	50.4	
4	Q9D8N0	Elongation factor 1-γ (Eef1g)	27.863	20	6	14	437	50	
4	Q3UX10	Tubulin α chain-like 3 (Tubal3)	30.405	8	4	89	446	50	
3	Q9CWF2	Tubulin β-2B chain (Tubb2b)	239.301	71	24	304	445	49.9	
3	P99024	Tubulin β-5 chain (Tubb5)	214.778	71	24	324	444	49.6	
3	Q9D8N0	Elongation factor 1-γ (Eef1g)	193.321	65	23	324	437	50	
3	P10126	Elongation factor 1-α1 (Eef1a1)	202.141	56	21	542	462	50.1	
3	Q9ERD7	Tubulin β-3 chain (Tubb3)	158.874	64	21	199	450	50.4	
3	P68369	Tubulin α-1A chain (Tuba1a)	188.056	64	20	177	451	50.1	Go et al. (2021)
3	P05213	Tubulin α-1B chain (Tuba1b)	178.724	64	20	169	451	50.1	
3	Q9D6F9	Tubulin β-4A chain (Tubb4a)	170.961	54	19	253	444	49.6	Go et al. (2021)
3	P62631	Elongation factor 1-α2 (Eef1a2)	50.614	28	9	259	463	50.4	
3	Q8BFR5	Elongation factor Tu, mitochondrial (Tufm)	39.656	32	9	28	452	49.5	
3	P60710	Actin, cytoplasmic 1 (Actb)	41.856	31	7	24	375	41.7	
3	P17182	α-Enolase (Eno1)	27.891	24	7	12	434	47.1	
3	P62737	Actin, aortic smooth muscle (Acta2)	24.601	16	5	16	377	42	
3	Q3UX10	Tubulin α chain-like 3 (Tubal3)	19.056	8	4	36	446	50	
3	P10637-3	Isoform Tau-B of Microtubule-associated protein tau (Mapt)	14.659	14	4	17	364	38.2	
2	Custom-P97783	Protein AF1q (Mllt11)	298.867	89	35	1363	320	36.7	
2	P48758	Carbonyl reductase [NADPH] 1 (Cbr1)	185.195	77	21	576	277	30.6	
2	P62908	40S ribosomal protein S3 (Rps3)	85.426	70	17	171	243	26.7	
2	P68040	Receptor of activated protein C kinase 1 (Rack1)	34.523	25	7	20	317	35.1	
2	O70251	Elongation factor 1-β (Eef1b)	16.545	24	4	9	225	24.7	
2	Q9D819	Inorganic pyrophosphatase (Ppa1)	10.238	15	3	6	289	32.6	
2	Q80XN0	D-β-hydroxybutyrate dehydrogenase, mitochondrial (Bdh1)	8.871	9	2	4	343	38.3	
1	P97783	Protein AF1q (Mllt11)	48.499	92	6	111	90	10	

To evaluate whether Mllt11 localized to growing neurites, we used immunocytochemistry to probe the subcellular distribution of Mllt11, Tubb3, and acetylated α-tubulin in cultures of primary fetal cortical neurons. Cortical neurons were extracted from wild-type embryos at E18.5 and cultured for 24 h or one week, then immunostained to reveal the localization of Tubb3, acetylated α-tubulin, and Mllt11. After 24 h *in vitro*, corresponding to the extension of a primary neurite, Mllt11/Tubb3 co-localized in discrete punctate patterns in varicosities along the distal portion of the developing neurite, as well as in the growth cone with limited overlap in the soma (Fig. 6*C*,*c'*) After one week in culture, neurons displayed a more elaborate neurite arborization pattern and acetylated α-tubulin and Mllt11 were both detected along proximal portion of neurite (Fig. 6*D*). *Mllt11* expression displayed a high degree of overlap with acetylated α-tubulin and β-tubulin isoforms differentially over neurite development, consistent with a role in neurite development. We next evaluated microtubule levels in the CC of *Mllt11* mutants and found that both Tubb3 and acetylated α-tubulin staining was reduced in cKOs at E18.5, reflecting reduced axonal targeting to the midline in the mutants (Fig. 6*E*).

### Mllt11 is required for neurite outgrowth and extension

**Figure 7.**
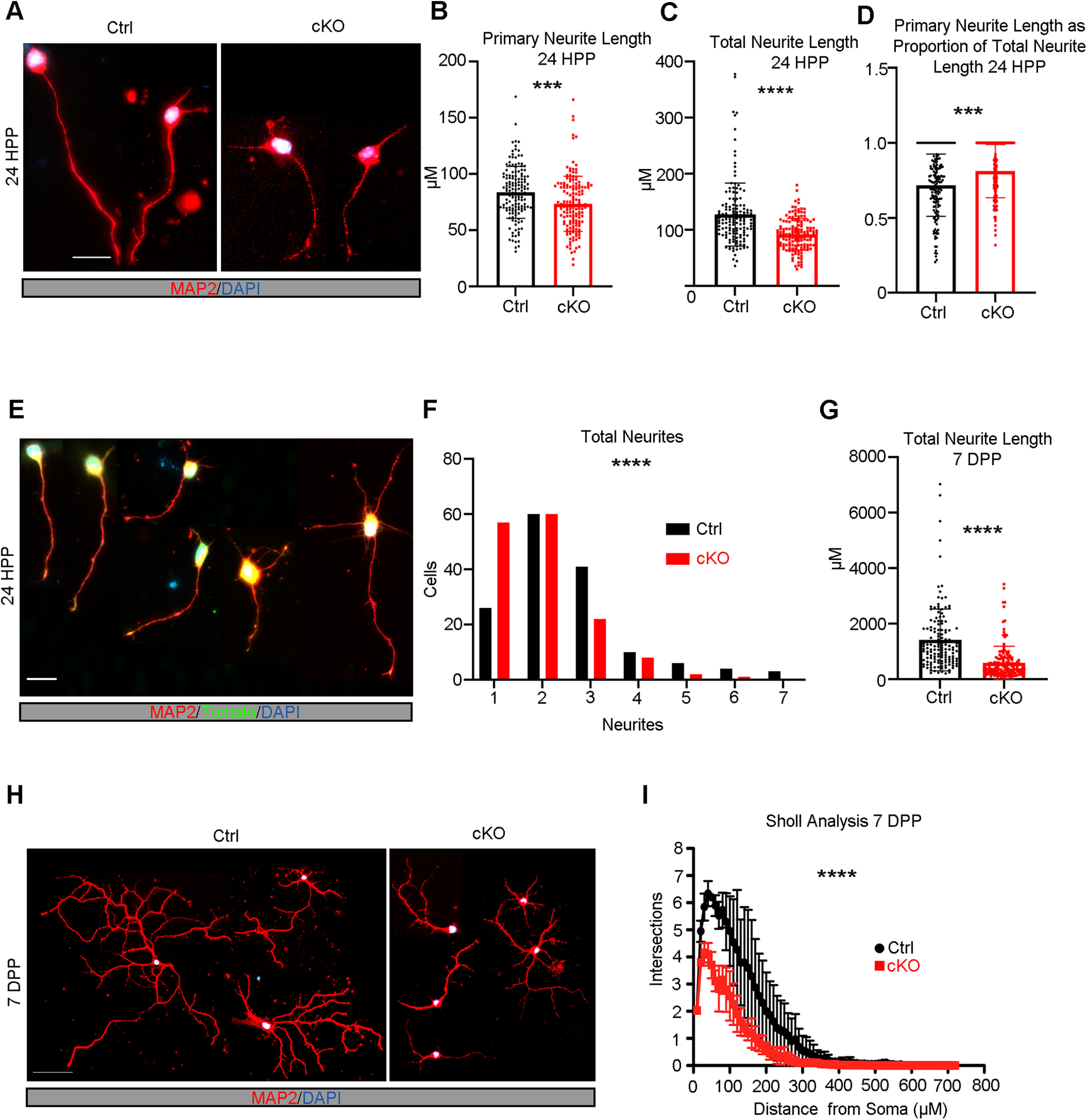
*Mllt11* loss decreased neurite outgrowth and branching complexity *in vitro*. ***A***, Primary cortical neurons derived from E18.5 brains cultured for 24 h postplating. Neurites identified with MAP2 staining (red). ***B***, ***C***, Twenty-four hours postplating, primary neurite length (***B***) and total neurite length (***C***) were significantly decreased in cKO neurons relative to controls (***D***). The proportion of total primary neurite length was significantly increased in cKO neurons relative to controls. ***E***, Examples of neuronal morphologies with varying numbers of neurites 24 h postplating. ***F***, Quantification of number of neurites per neuron 24 h postplating, where cKO neurons had significantly fewer neurites compared with controls. ***G–I***, Quantification of decreases in total neurite length (***G***) and branching complexity (***I***) of cKO relative to control primary cortical neurons cultured for 7 d postplating (***H***). Student's *t* test with Welch's correction (***B–D***, ***F***). Paired *t* test, for both controls and cKOs *N* = 150 neurons (50 neurons/individual × three individuals). Data presented as mean ± SD; *p ≤ 0.05, **p ≤ 0.01, ***p ≤ 0.001, ****p ≤ 0.0001. Scale bar: 20 μm (***A***, ***E***) and 50 μm (***H***). HPP, hours postplating; DPP, days postplating.

The relationship between stabilized and dynamic forms of tubulin has been shown to regulate the migratory potential of neurons and neurite extension because of the cycling of microtubule severing during outgrowth (Sudo and Baas, 2010; Lin and Smith, 2015; Wei et al., 2018). If *Mllt11* loss affected the relative amounts of stabilized microtubules, we would expect to see significant changes in neurite morphogenesis of UL neurons. We therefore evaluated the morphology of cultured primary cortical neurons from *Mllt11* cKO mutants. We took advantage of the Ai9 tdTomato+ reporter introduced into the *Cux2iresCre/*+*; Mllt11*^*Flox*/+^ conditional mouse to efficiently label and isolate UL primary neurons in culture. After 24 h *in vitro*, the length of MAP2+ primary neurites was reduced in cKOs compared with controls (Fig. 7*A*,*B*), as was the total length of all neurites (Fig. 7*C*). Moreover, the proportion of total length of extended neurites represented by the primary neurite at 24 h was slightly increased in cKOs (Fig. 7*D*). Quantification of the average number of neurites per neuron revealed a significant decrease in cKOs compared with controls (Fig. 7*E*,*F*), further suggesting that initiation of neurite outgrowth was abrogated on *Mllt11* loss.

After one week of culture *in vitro*, the total neurite length was drastically reduced in *Mllt11* cKO CPNs (Fig. 7*G*). Since *Mllt11* loss progressively attenuated primary neurite outgrowth in cultured primary UL CPNs, we next evaluated a requirement for Mllt11 in the elaboration of dendritic arborization patterns in cultured CPNs. An advantage of using cultured neurons is that they are free of apical and basal contacts, allowing their neurites to radiate uniformly from the soma. This feature of two-dimensional neuronal culture allows for the evaluation of dendritic complexity by Sholl analysis. *Mllt11* mutant primary neurons displayed much less complexity in the branching of their neurites (Fig. 7*H*,*I*), thus *Mllt11* loss greatly impacted the arborization patterns of cultured UL CPNs.

**Figure 8.**
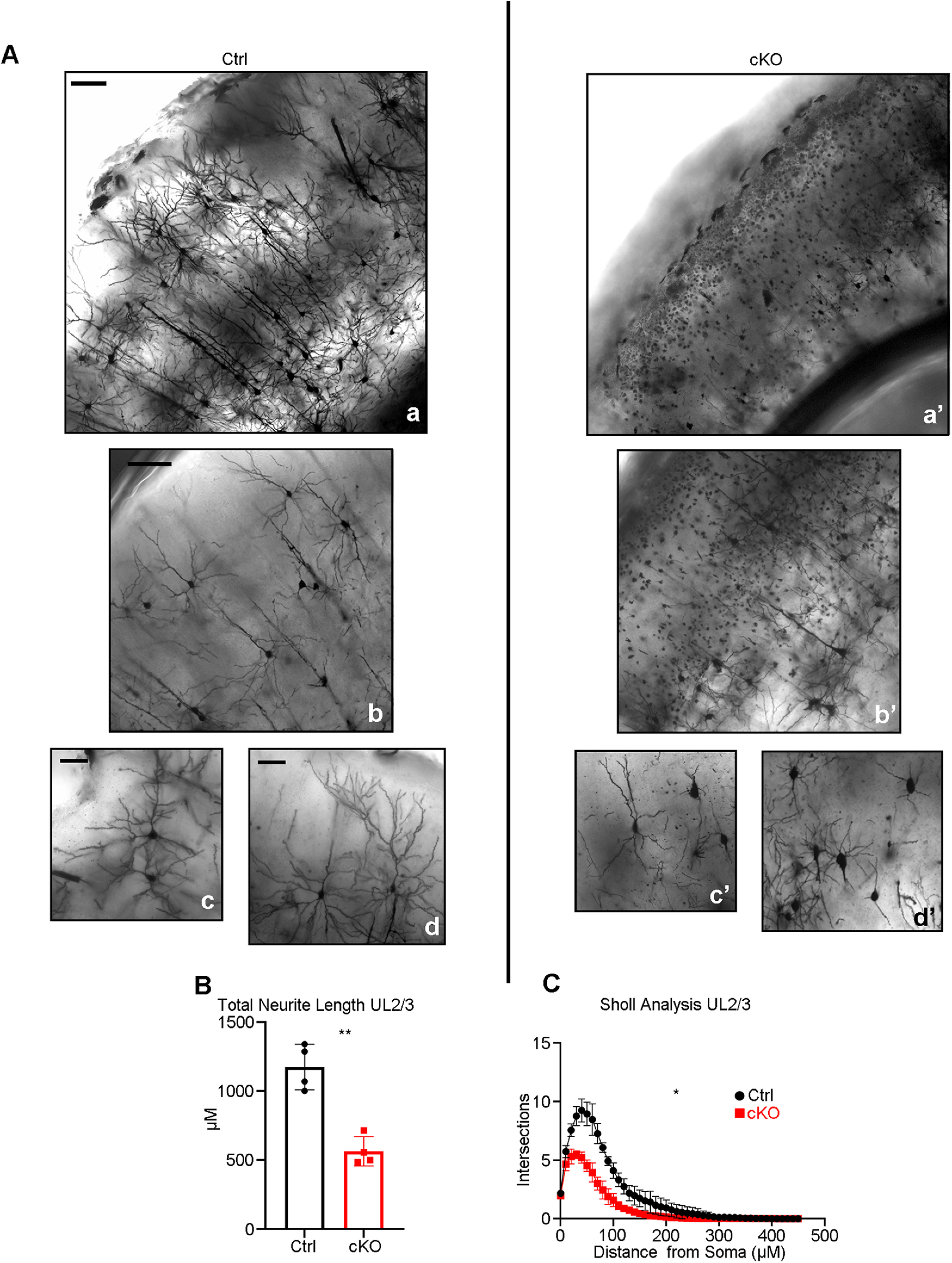
Decreased neurite outgrowth and branching complexity in UL CPNs in the *Mllt11* cKO cortex. ***A***, Corresponding 100× (***Aa***, ***Aa'***) and 200× (***Ab–Ad'***) magnification images of coronal sections of P28 Golgi-stained control (***Aa–Ad***) and *Mllt11* cKO cortices (***Aa'–Ad'***). ***B***, ***C***, Quantification of neurite length (***B***) and branching complexity (***C***) within UL2/3 showed reduced neurite length and branching complexity in cKOs compared with controls. Length measurements analyzed with Student's *t* test with Welch's correction, layer-specific Sholl analysis compared with paired *t* test, *N* = 4 (controls and cKOs), 30 neurons/individual. Data presented as mean ± SD; *p ≤ 0.05, **p ≤ 0.01. Scale bar: 100 μm (***Aa***, ***Aa'***) and 50 μm (***Ab–Ad'***).

Given the prevalence of neurite growth and pruning during postnatal development, we evaluated the requirement of Mllt11 in the formation of dendritic arborization morphology characteristic of UL CNs *in vivo* at a time when mature arborization patterns have fully developed. Golgi staining was performed on control and *Mllt11* cKO brains harvested at P28 to capture mature CPN morphologies using the Golgi staining method. While control UL CPNs displayed complex dendritic morphologies typical of cortical pyramidal neurons (Fig. 8*Aa–Ad*), *Mllt11* cKOs UL CPNs displayed greatly attenuated dendritic arborization morphologies (Fig. 8*Aa'–Ad'*). We quantified the length of neurites in the Golgi-stained UL CPNs of cKO and control brains and observed a severe decrease in total neurite length in the *Mllt11* mutants (Fig. 8*B*). The extent of decrease of neurite arborization *in vivo* was similar to that observed in cultured primary neurons (Fig. 7*G–I*). Sholl analysis revealed that the overall neuronal morphology was also much less complex with less elaborately-branched neurites in *Mllt11* cKO UL CPNs (Fig. 8*C*). Taken together, these findings confirmed a critical requirement of Mllt11 in the growth and elaboration of neurites during the development of mature superficial CPN morphologies and projection phenotypes.

## Discussion

We described the role of a novel vertebrate-specific and neuronally restricted protein, Mllt11, in the regulation of neurite extension and migration of superficial CPNs. The *Mllt11* locus has been identified in human genomic regions potentially affected in schizophrenia patients (Brzustowicz et al., 2002, 2004; Su et al., 2017), suggesting a role in CNS development. Our study is the first to demonstrate that Mllt11 regulates key aspects of cortical neuron morphogenesis and connectivity. Using the *Cux2iresCre* driver to excise *Mllt11* in developing UL CPNs, we revealed that conditional *Mllt11* loss decreased cortical WM tracts and callosal fibers, and abrogated extension and maintenance of mature arborescent dendrites. Furthermore, *Mllt11* conditional mutants did not affect the formation of Reelin+ CR cells nor radial glial scaffolding, both of which are crucial for guiding the migration of CPNs. This suggests that *Mllt11* loss acted cell autonomously to hinder radial migration of CPN progenitors into the CP. The inefficient migration of UL CPNs in *Mllt11* mutant brains likely reflected a role for Mllt11 in regulating the cytoskeleton, as it associated with stabilized microtubules, and was required for their maintenance in growing neurites. Neurons lacking *Mllt11* displayed deficits in neurite complexity both *in vivo* and in *vitro*, demonstrating that Mllt11 is a critical regulator of neurite outgrowth.

We show that Mllt11 interacts with tubulin isoforms, confirming a recent proteomic screen (Go et al., 2021), which provides a molecular rationale for the basis of the *Mllt11* mutant phenotype. Cortical thinning and increased ventricular lumen surface area are common phenotypes associated with neurodevelopmental disorders, and were observed in *Mllt11* mutant brains, suggesting cytoskeletal dysregulation may underlie a subset of these disorders (Bahi-Buisson et al., 2014; Mensen et al., 2017; Li et al., 2019). Moreover, changes in microtubule dynamics can affect neuronal migration and the generation of functional connections in the brain. These are processes whose dysfunction may underlie a wide spectrum of neurodevelopmental disorders. Given that *Mllt11* mutant mice displayed both migratory and neurite outgrowth defects, they are a model to study brain disorders arising from aberrant regulation of the neuronal cytoskeletal during development.

The microtubule cytoskeleton generates polarized force and provides a functional railway along which motor proteins migrate, whether to drive actin polymerization in generating protrusive forces at the leading edge of the growth cone, or integrate extracellular cues into regulation of microtubule activity (Dent and Kalil, 2001; Buck and Zheng, 2002; Lee et al., 2004; Zhou et al., 2004). Tubulin mutations have been shown to impair neuronal migration into the cortex (Aiken et al., 2017), and specifically affect UL CPNs (Keays et al., 2007) reliant on dynamic, multipolar cytoskeletal reorganization to extend short processes, “crawling” through established DL CPNs (Miyata et al., 2001; Sakakibara et al., 2014). The dynamic expression of *Mllt11* during UL CPN development also supports its role as a critical regulator of UL migration. Interestingly, mutations in α-tubulin have been shown to cause axonal trafficking defects that impair synaptic stability and function without impacting axonal degeneration or neuronal survival (Buscaglia et al., 2020). The interaction of Mllt11 with microtubules could therefore provide a mechanistic explanation for the impaired migration and reduced CPN dendritic complexity seen in *Mllt11* mutants. Specifically, we showed that Mllt11 associates with acetylated α-tubulin in fetal brain lysates, suggesting that it may be required for the stabilization of the cytoskeleton in growing neurites, and account for the similarities between the *Mllt11* mutant phenotype with those targeting tubulin isoforms.

The inefficient extension of neurites may also be attributed to impaired trafficking of organelles and ribosomes for local protein synthesis, or vesicles carrying secreted cues and growth cone machinery to the axonal periphery (Kennedy and Ehlers, 2006; González et al., 2016). Specifically, a potential Mllt11 interactor Myosin 10 (Table 1) is known to regulate radial migration of CPNs through its effect on the localization of N-cadherin (Lai et al., 2015), and has been implicated in tumor invasion by regulating process extension (Ropars et al., 2016). It is tempting to speculate that altered neuronal trafficking may underlie morphologic and transcriptional deficits of *Mllt11* mutant CPNs. Loss of *Mllt11* from the superficial cortex altered levels of UL2/3-specific transcription factors Satb2 and CDP/Cux1, which function in establishment and maintenance of the CC as well as mature arborescent neuronal morphology and somal packing (Alcamo et al., 2008; Cubelos et al., 2015; Rodríguez-Tornos et al., 2016; He et al., 2021). Importantly, the loss of layer-specific morphology and identity has been demonstrated in postmortem brains from autistic humans (Casanova et al., 2013; Stoner et al., 2014; Fujimoto et al., 2021). These clinical data are reminiscent of the severe dysplasia in the cortex of our conditional *Mllt11* mutants, revealed by the Golgi staining method. It is presently unclear whether the loss of UL-specific gene expression we observed in our *Mllt11* mutants, and tubulinopathies more generally, is primarily because of cytoskeletal dysregulation, or other uncharacterized gene expression pathways functioning downstream of Mllt11. Some neuronal subtypes exhibit altered expression of subpopulation-specific markers when cytoskeletal regulation is altered such that connections to target regions cannot be established (Hippenmeyer et al., 2005), implying that there may be uncharacterized feedback mechanisms between establishment of connectivity and maintenance of expression of CPN subtype-specific transcription factors.

Other potential roles for Mllt11 may depend on binding to chaperon protein HSPa8 and regulating protein export from the nucleus required for protein degradation (Li et al., 2014). Yet another possible function for Mllt11 may include regulating the Wnt signaling cascade via T cell factor 7, which in turn regulates CD44 to promote cell migration and metastasis (Park et al., 2015; Li et al., 2018). As ours is the first study to explore Mllt11 function in the developing brain, additional studies are needed to determine whether Mllt11 could be exerting its effect on transcriptional regulation directly or indirectly via cytoskeletal interactions and intracellular trafficking, or both.

In light of this, we showed that the overexpression of *Mllt11* promoted migration into the CP, consistent with a cytoskeletal regulatory function. This finding is also consistent with *in vitro* studies, which showed that the overexpression of *Mllt11* in cancer stem cell lines promotes proliferation and invasiveness (Tse et al., 2017). It is possible that Mllt11-dependent cytoskeleton re-organization may favor symmetric divisions of organelles, nuclear components, and polarity protein complexes, therefore enhancing invasiveness of cells (Nance and Zallen, 2011; Piroli et al., 2019). On the other hand, the transcriptional regulation of Mllt11 by REST has been associated with promoting neuronal differentiation, with high REST and low Mllt11 expression in undifferentiated neuronal tissue, and increasing Mllt11 levels correlated with decreased REST activity during terminal neuronal differentiation (Hu et al., 2015). These previously published findings, in conjunction with the complexity of the *Mllt11* mutant phenotype we reported here, imply that there are potentially two discrete pathways through which Mllt11 regulates neuronal development: a cytoskeletal regulatory mechanism, and a transcriptional regulatory mechanism.

Much of what is known about Mllt11 has been discerned from overexpression studies in immortalized cell lines and oncogenic clinical case studies, revealing pathogenic roles in cell process extension, invasiveness, and secretion of factors that promote efficient cellular motility (Lin et al., 2004; Tse et al., 2004; Li et al., 2006; Chang et al., 2008; Tiberio et al., 2017; Park et al., 2019). Oncogenesis and neurogenesis share commonalities at the level of cytoskeletal regulation of somatic and nuclear morphology, as well as extension of and trafficking along cytoplasmic processes, allowing for invasion of and migration through tissues. However, it is important to make the distinction between pathologic studies and our current findings on the role of Mllt11. Until the current study, the physiological role for Mllt11 was unclear. In all the published *in vitro* and oncogenic case studies, Mllt11 is aberrantly overexpressed in non-neuronal tissues, either by itself or as a fusion with the chromatin remodeling protein Mll. We previously reported that Mllt11 protein and mRNA was exclusively localized to the developing CNS, and not in any other tissues (Yamada et al., 2014). The expression pattern of β-gal in the targeted *Mllt11* allele confirmed that it is normally expressed only within the developing CNS, with the highest levels reflecting UL cortical neurogenesis. Consistent with this expression profile, we now show that Mllt11 is required for proper migration but not neurogenesis of UL CPNs. Furthermore, we also revealed that Mllt11 regulates neurite outgrowth and the maintenance of UL gene expression programs. The loss of *Mllt11* led to a reduction of interhemispheric connectivity *via* reduced crossing callosal fibers. We also characterized a severe dysplasia of CPNs in *Mllt11* cKO mutant neonatal brains; a phenotype found in severe neurodevelopmental disorders such as ASD and Fragile X-associated tremor ataxia syndrome with which Mllt11 dysregulation has been associated (Xu et al., 2016; Drozd et al., 2019). Finally, we provide a possible mechanism of action for Mllt11 to link these phenotypes via its association with microtubules.

In summary, we investigated the role of Mllt11 in development of UL CPNs using a genetic KO and labeling strategy to target the superficial cortex. Numbers, laminar distribution, and morphology of UL CPNs were assessed over development. By combining *in vitro* and *in vivo* neurite outgrowth and morphology assays, we demonstrate that Mllt11 is required for neuronal invasion in the CP, formation of mature dendritic morphologies characteristic of UL CPNs, and the extension of axons across the CC. Mllt11 interacts with the microtubule cytoskeleton and likely exerts its effect by altering cytoskeletal organization during development. Whether the regulatory role of *Mllt11*is exerted through the stabilization of cytoskeletal architecture or by trafficking of cellular machinery along neurites will be the subject of future investigations.
