# Supplementary figures and images for: Mllt11 Regulates Migration and Neurite Outgrowth of Cortical Projection Neurons during Development
Source: J Neurosci. 2022 May 11;42(19):3931–48. doi: 10.1523/JNEUROSCI.0124-22.2022 (PMC9097781; doi:10.1523/JNEUROSCI.0124-22.2022)

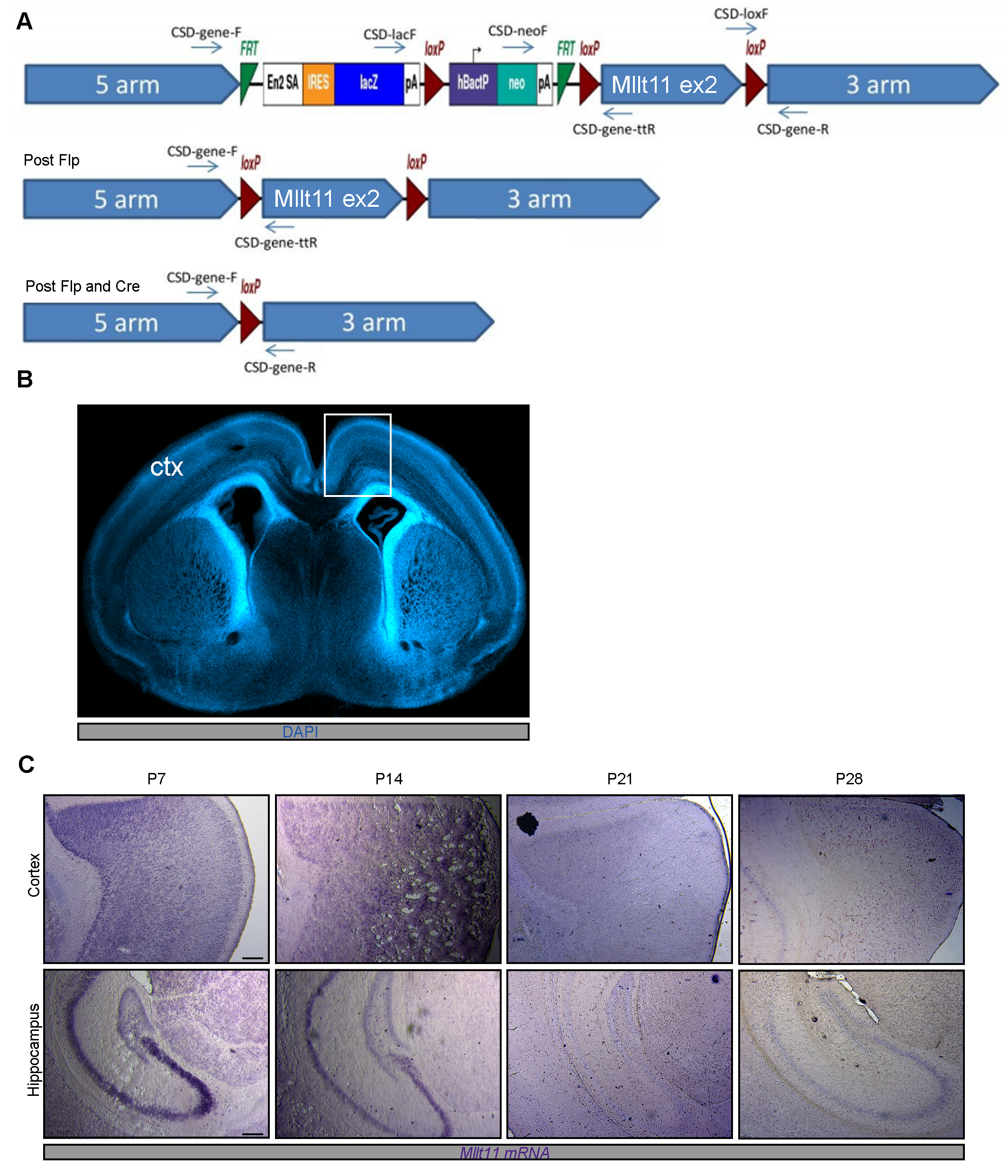

Supplement: Extended Data Figure 1-1 — Mllt11 targeting strategy and ontogenic expression profile. A, Graphic representation of the targeting construct inserted into the Mllt11 locus before and after flp and cre recombinase activity. The entire protein-coding region of Mllt11 is encoded by exon 2 (Mllt11 ex2), which is flanked by loxP sites. Mllt11 expression can be evaluated by β-Gal staining because of the insertion of lacZ cDNA in the targeted allele. A cKO allele can be garneted by the removal of the lacZ and selection cassette by germline Flp recombination. B, A reference coronal section of a control brain with a boxed region to indicate the area sampled in panel C. C, Mllt11 expression in the cortex (top panel) and hippocampus (bottom panel) from P7 to P28. RNA levels declined in the cortex were indistinguishable from background at P28. Scale bar: 100 μm. Download Figure 1-1, TIF file. [file ns-JN-RM-0124-22-s01.tif]

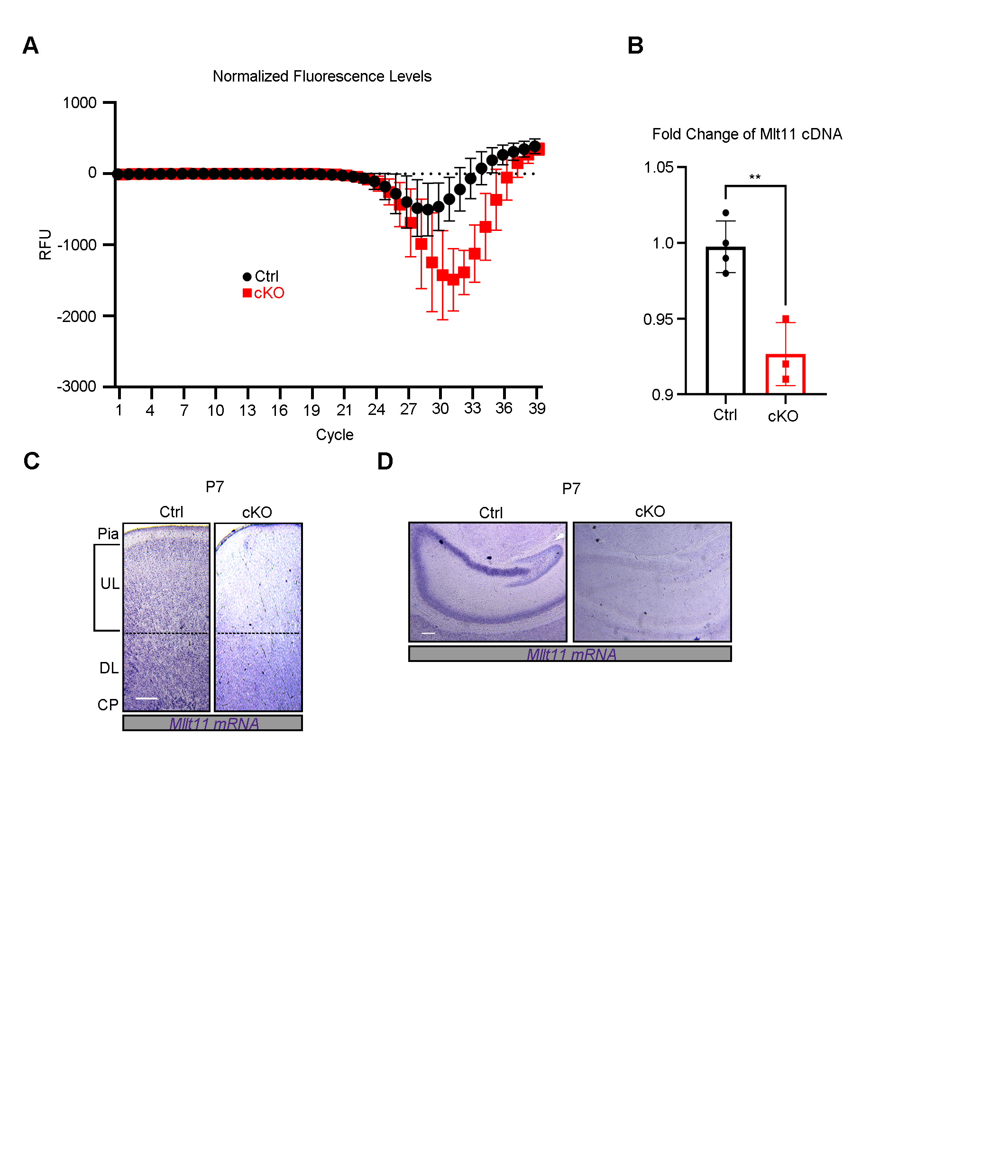

Supplement: Extended Data Figure 1-2 — Mllt11 cKO validation by qPCR and ISH. A, Quantitation of qPCR fluorescence levels of control and cKO cortices normalized to internal control GAPDH. B, Fold change of Mllt11 cDNA transcript levels was significantly decreased in cKO relative to control brains. C, D, Images of ISH of Mllt11 riboprobe on P7 control and cKO cortices (C) and hippocampi (D) showed decreased labeling in the superficial cortex, corresponding to the Cux2-expressing region. Student's t test with Welch's correction, (A, B) N = 4 controls, 3 cKOs, (C, D) N = 3. Data presented as mean ± SD; **p ≤ 0.01. Scale bar: 100 μm. CP, cortical plate; RFU, relative fluorescence units. Download Figure 1-2, TIF file. [file ns-JN-RM-0124-22-s02.tif]

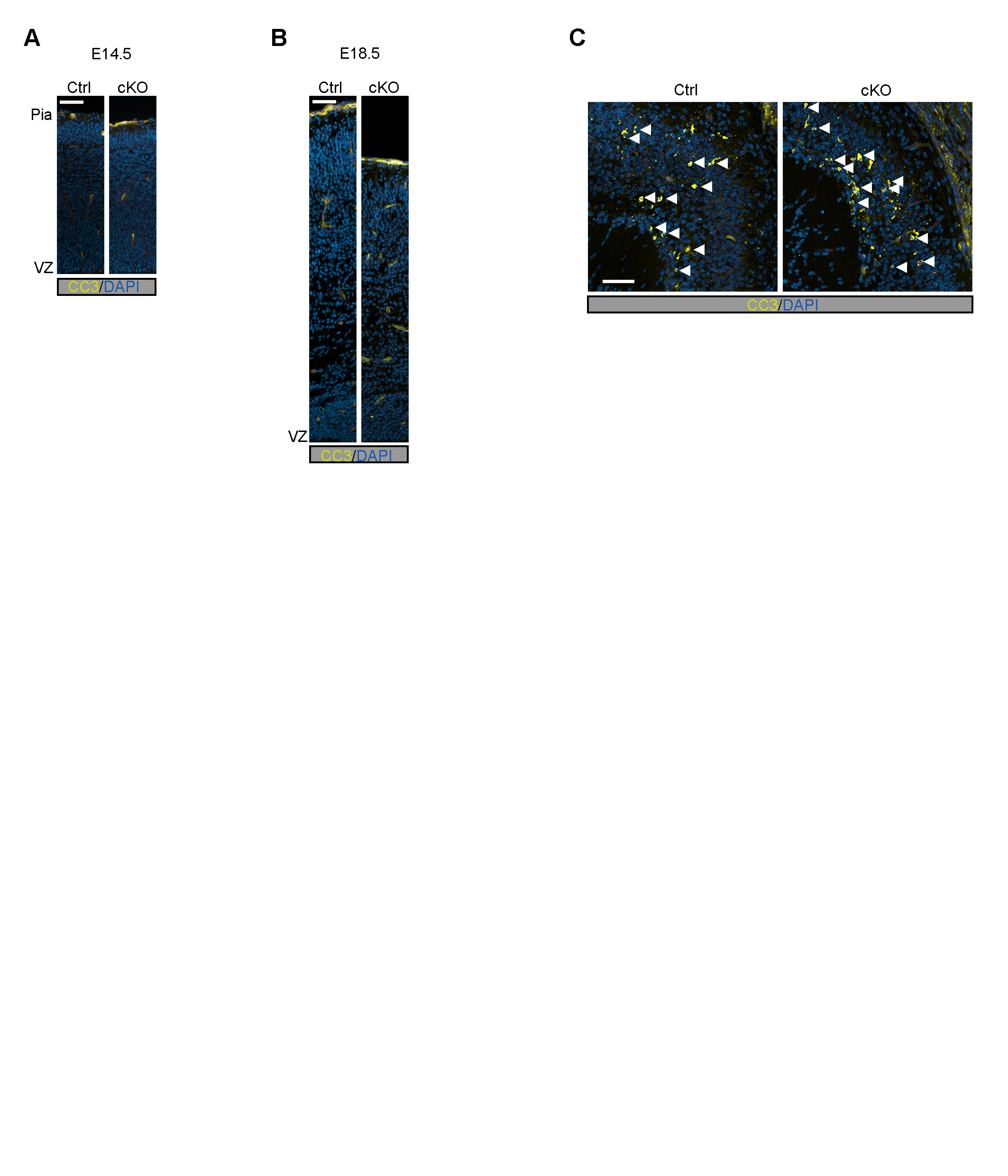

Supplement: Extended Data Figure 1-3 — Loss of Mllt11 had no impact on programmed cell death. A, B, Cortical slices at E14.5 (A) and E18.5 (B) showed no differences in the levels of CC3 in cKOs relative to controls. C, CC3 staining in the retrosplenial area, which normally has enhanced apoptosis, was included as an antibody control for CC3 staining. White arrowheads indicate positive labeling. N = 3 controls, 5 cKOs. Scale bar: 50 μm. VZ, ventricular zone. Download Figure 1-3, TIF file. [file ns-JN-RM-0124-22-s03.tif]

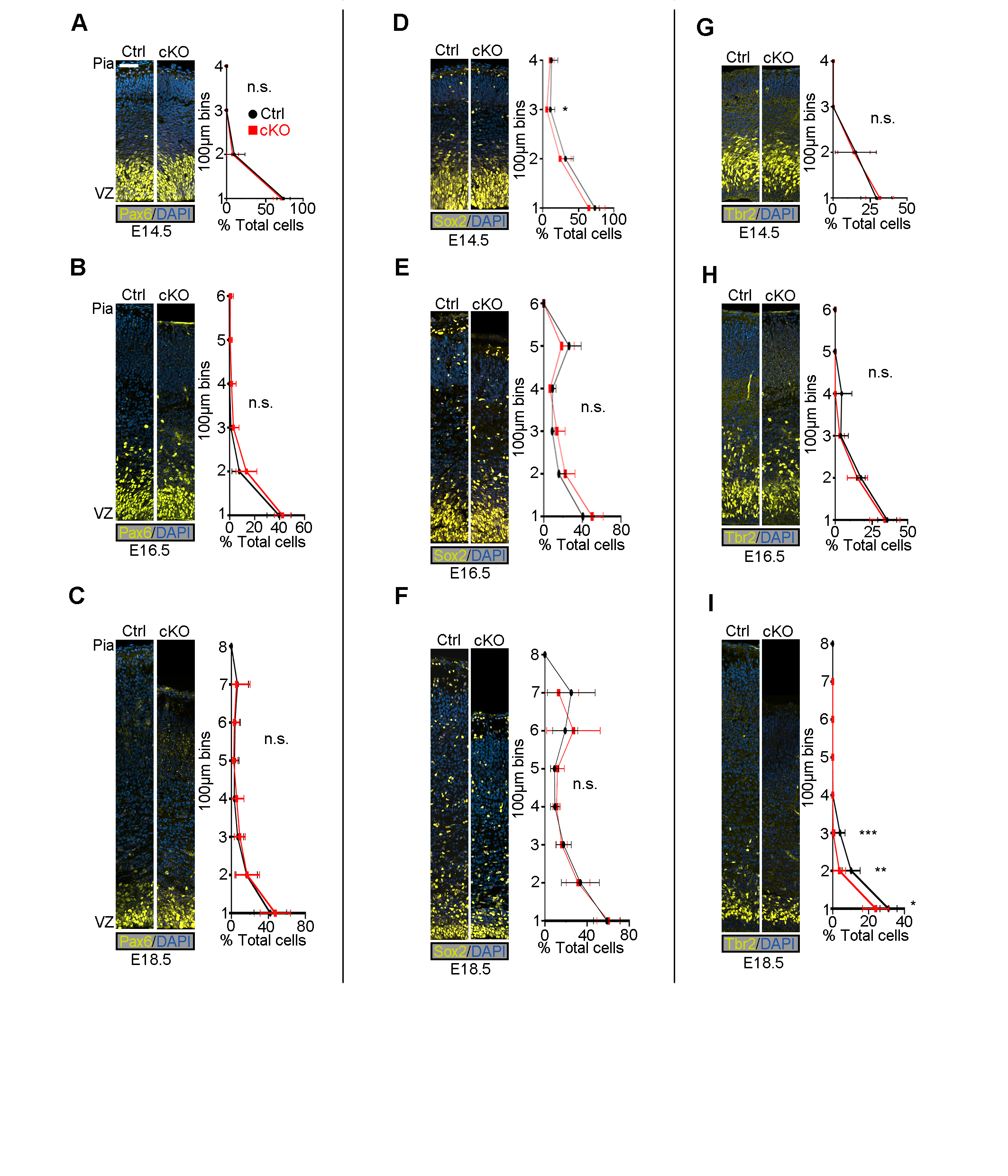

Supplement: Extended Data Figure 1-4 — Neural progenitor populations were largely unaffected in Mllt11 cKO mutants. A–C, Coronal cortical slices stained for Pax6 with IHC was unaltered in cKO cortices relative to controls at E14.5 (A), E16.5 (B), and E18.5 (C). D–F, Sox2 levels were largely similar between cKO and controls at E14.5 (D), E16.5 (E), and E18.5 (F). G–I, Tbr2 expression was also largely unaltered in cKOs at E14.5 (G) and E16.5 (H), but showed a significant trend toward decreased levels normalized to DAPI+ nuclei immediately above the Tbr2+ progenitor domain at E18.5 (I). Line charts represent percentage of positive cells normalized to DAPI+ nuclei per 100 × 100 μm bin. Student's t test with Welch's correction, (A–C, F–I) N = 4, (D, E) N = 3. Data presented as mean ± SD n.s., not significant; *p ≤ 0.05, **p ≤ 0.01, ***p ≤ 0.001. Scale bar: 50 μm (A–I). VZ, ventricular zone. Download Figure 1-4, TIF file. [file ns-JN-RM-0124-22-s04.tif]

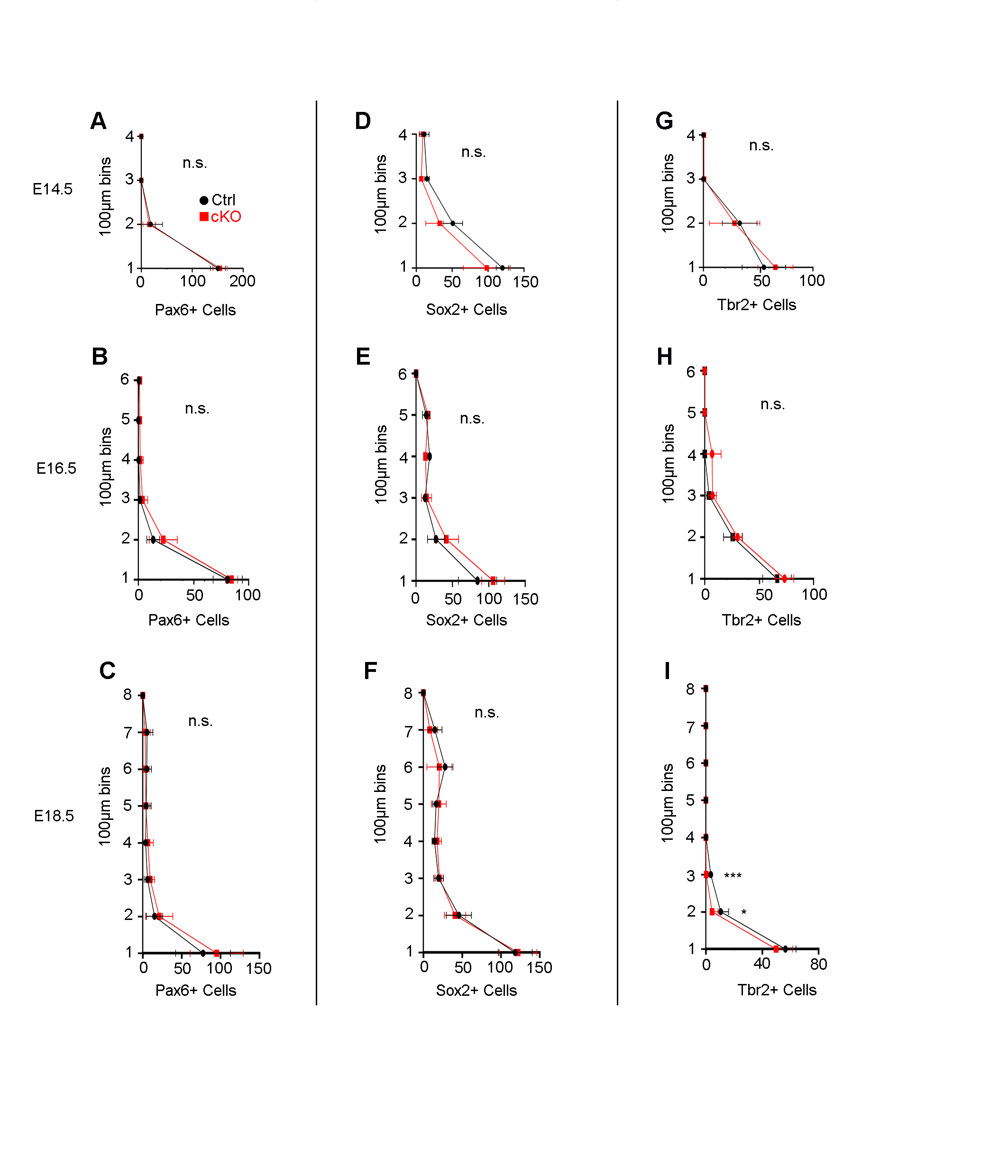

Supplement: Extended Data Figure 1-5 — Total cell numbers of neuronal progenitors in control and Mllt11 cKO cortices. A–C, Distribution of total Pax6+ cells in control (black lines) and Mllt11 cKO (red lines) at E14.5 (A), E16.5 (B), and E18.5 (C) showing comparable levels at all stages. D–F, Distribution of total Sox2+ cells at E14.5 (D), E16.5 (E), and E18.5 (F) showing comparable levels at all stages. G–I, Distribution of total Tbr2+ cells at E14.5 (G), E16.5 (H), and E18.5 (I) showing comparable levels at E14.5 (G) and E16.5 (H), and slightly decreased levels immediately above the Tbr2+ progenitor domain in cKOs at E18.5 (I). Line charts represent total cells positive for each marker per 100 × 100 μm bin. Student's t test with Welch's correction, (A–C, F–I) N = 4, (D, E) N = 3. Data presented as mean ± SD n.s., not significant; *p ≤ 0.05, **p ≤ 0.01, ***p ≤ 0.001. Download Figure 1-5, TIF file. [file ns-JN-RM-0124-22-s05.tif]

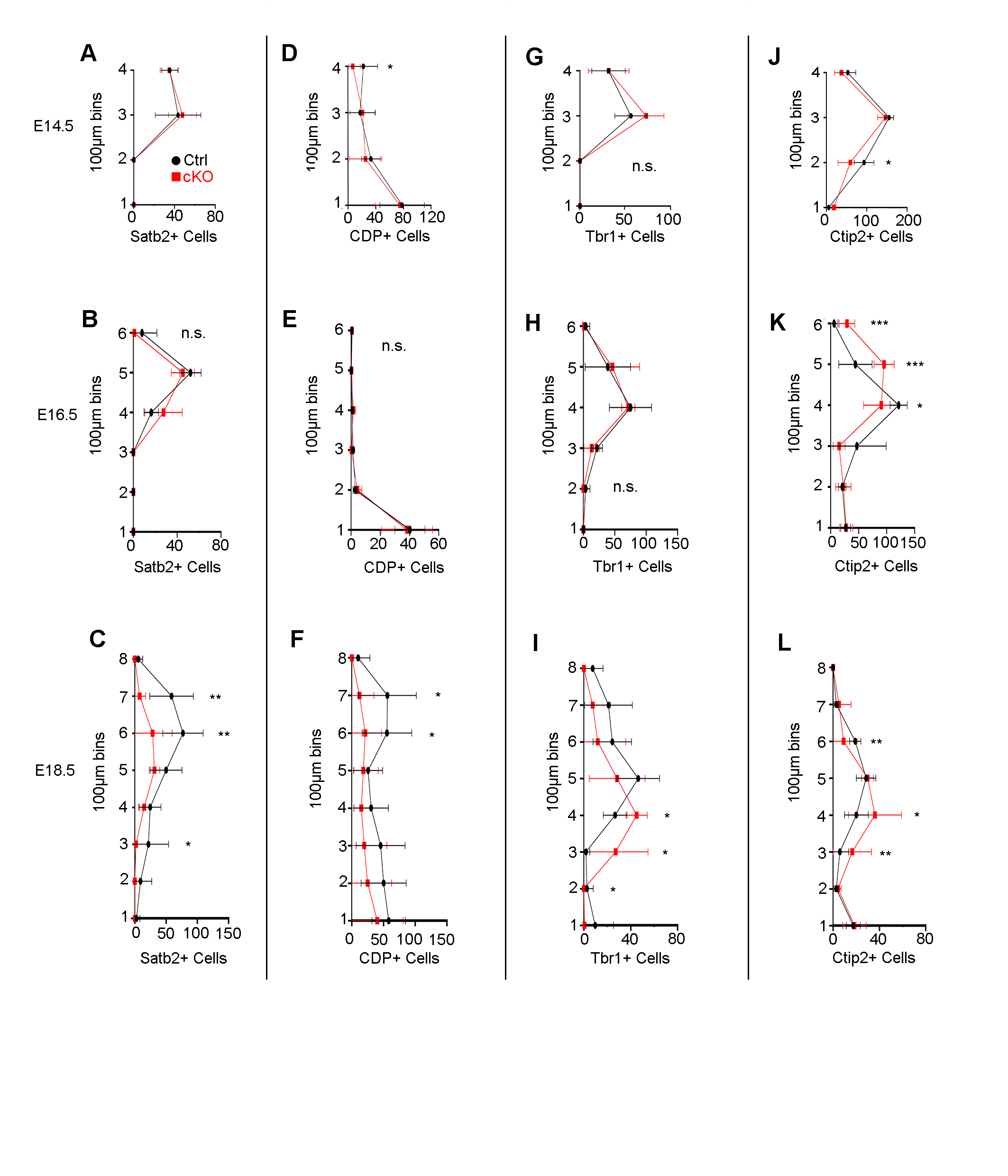

Supplement: Extended Data Figure 2-1 — Total cell numbers of cortical layer markers in control and Mllt11 cKO cortices. A–C, Distribution of total Satb2+ cells in control (black lines) and Mllt11 cKO (red lines), with comparable levels at E14.5 (A) and E16.5 (B), but a significant decrease in the mutant cortex at E18.5 (C). D–F, Distribution of total CDP+ cells with comparable levels at E14.5 (A) and E16.5 (B) and a decrease in mutants compared at E18.5 (F). G–I, Distribution of total Tbr1+ cells in the cortex showing comparable levels at E14.5 (G) and E16.5 (H), with mutants exhibiting an apical shift in expression domain at E18.5 (I). J–L, Distribution of total Ctip2+ cells in the cortex showing comparable levels at E14.5 (J), and an apical shift in expression domain beginning at E16.5 (K) and persisting at E18.5 (L) in Mllt11 cKO mutants compared with controls. Line charts represent total cells positive for each marker per 100 × 100 μm bin. Student's t test with Welch's correction, (A, E, G, I–L) N = 4, (F) N = 5, (H) N = 4 controls, 5 mutants. Data presented as mean ± SD n.s., not significant; *p ≤ 0.05, **p ≤ 0.01, ***p ≤ 0.001. Download Figure 2-1, TIF file. [file ns-JN-RM-0124-22-s06.tif]

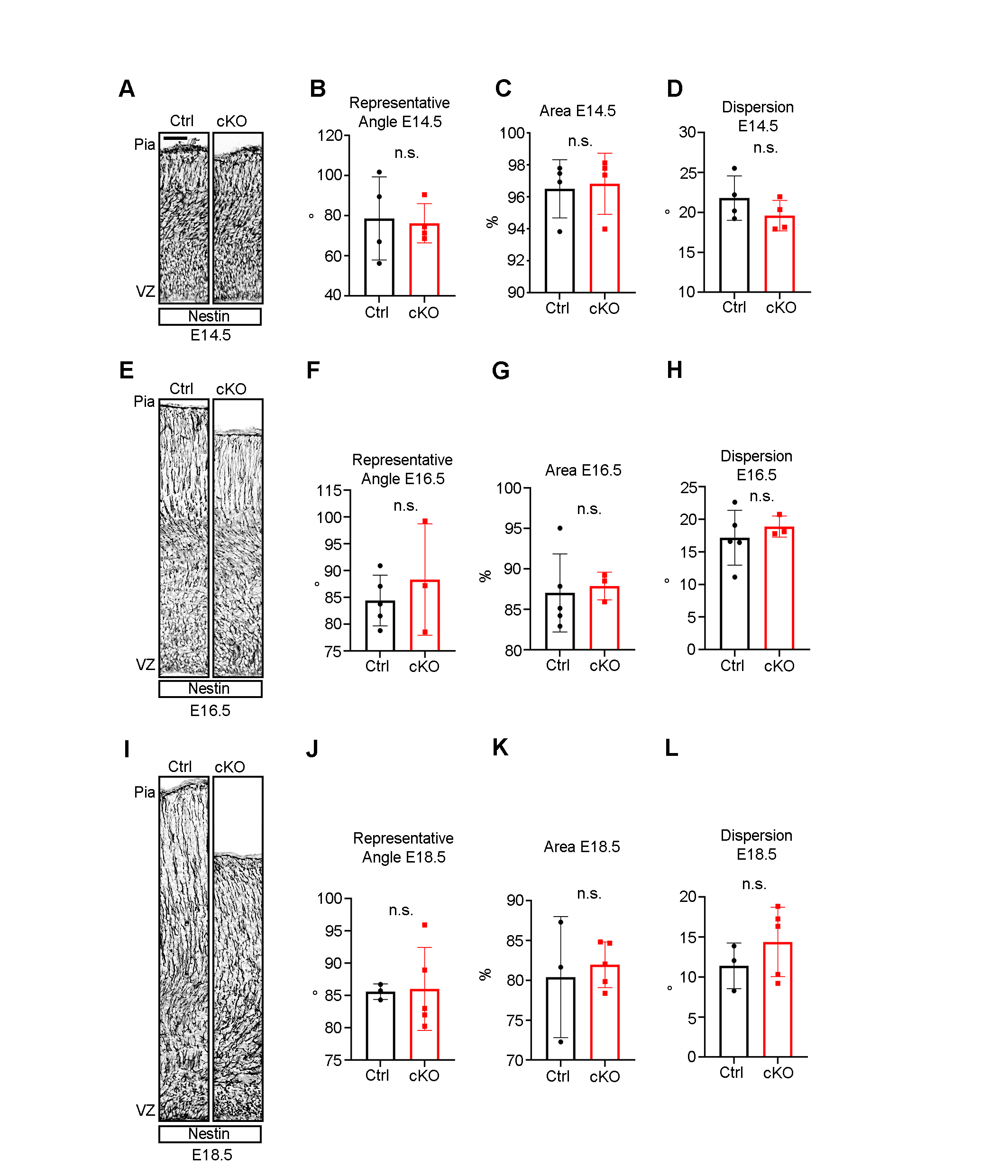

Supplement: Extended Data Figure 3-1 — Cortical radial glia were unaltered in Mllt11 cKOs. A, Coronal cortical slices at E14.5 showing Nestin immunostainiong in control versus Mllt11 cKO mutants. B–D, Representative angle (B), area (C), and fiber dispersion (D) showed no significant differences between controls and cKOs. D, Nestin expression in the cortex at E16.5. E–G, Representative angle (E), area (F), and dispersion (G) showed no significant differences between control and cKO cortices at E16.5. I, Nestin staining at E18.5. J–L, Representative angle (J), area (K), and fiber dispersion (L) show no significant differences between controls and cKOs at E18.5. Student's t test with Welch's correction, (A–D) N = 4, (E–H) N = 5, (I–L) N = 3. Data presented as mean ± SD n.s., not significant. Scale bar: 50 μm (A, E, I). VZ, ventricular zone. Download Figure 3-1, TIF file. [file ns-JN-RM-0124-22-s07.tif]
